# Mitochondrial Proteome Changes in Rett Syndrome

**DOI:** 10.3390/biology12070956

**Published:** 2023-07-03

**Authors:** Gocha Golubiani, Laura van Agen, Lia Tsverava, Revaz Solomonia, Michael Müller

**Affiliations:** 1 Institut für Neuro- und Sinnesphysiologie, Georg-August Universität Göttingen, Universitätsmedizin Göttingen, D-37073 Göttingen, Germany; 2 Institute of Chemical Biology, Ilia State University, Tbilisi 0162, Georgia; 3 Ivane Beritashvili Centre of Experimental Biomedicine, Tbilisi 0160, Georgia

**Keywords:** mitochondria, hippocampus, neocortex, *Mecp2*, proteomics, mouse model, Rett syndrome

## Abstract

**Simple Summary:**

Rett syndrome (RTT) is a genetic disorder caused by mutations in the X-chromosome. These mutations distort the function of a protein (methyl-CpG-binding protein 2), which controls the expression of several other genes. The resulting pathology, which manifests mostly in female patients, is associated with a number of deficits in brain functioning and development. On the cellular level, the defective functioning of mitochondria—the powerplants of the cells—is one of several hallmarks of RTT. Mitochondria contain more than 1000 different proteins, and their composition differs among tissues. Our study aims to reveal differences between the mitochondrial proteomes of a mouse model of RTT and wild-type (WT) mice. Two brain regions were studied, the hippocampus and neocortex, both of which are pivotal for cognitive function. Indeed, numerous differences were identified, which are not only mouse strain (genotype)-specific but also brain-region-specific. These differentially expressed proteins could affect mitochondrial dynamics, oxidative phosphorylation, and other important aspects of mitochondrial functioning. The data obtained will contribute to improving our understanding of mitochondrial malfunctioning during the progression of RTT, and they will pave the way for further translational medicine research.

**Abstract:**

Rett syndrome (RTT) is a genetic neurodevelopmental disorder with mutations in the X-chromosomal *MECP2* (methyl-CpG-binding protein 2) gene. Most patients are young girls. For 7–18 months after birth, they hardly present any symptoms; later they develop mental problems, a lack of communication, irregular sleep and breathing, motor dysfunction, hand stereotypies, and seizures. The complex pathology involves mitochondrial structure and function. *Mecp2^−/y^* hippocampal astrocytes show increased mitochondrial contents. Neurons and glia suffer from oxidative stress, a lack of ATP, and increased hypoxia vulnerability. This spectrum of changes demands comprehensive molecular studies of mitochondria to further define their pathogenic role in RTT. Therefore, we applied a comparative proteomic approach for the first time to study the entity of mitochondrial proteins in a mouse model of RTT. In the neocortex and hippocampus of symptomatic male mice, two-dimensional gel electrophoresis and subsequent mass-spectrometry identified various differentially expressed mitochondrial proteins, including components of respiratory chain complexes I and III and the ATP-synthase FoF1 complex. The NADH-ubiquinone oxidoreductase 75 kDa subunit, NADH dehydrogenase [ubiquinone] iron-sulfur protein 8, NADH dehydrogenase [ubiquinone] flavoprotein 2, cytochrome b-c1 complex subunit 1, and ATP synthase subunit d are upregulated either in the hippocampus alone or both the hippocampus and neocortex of *Mecp2^−/y^* mice. Furthermore, the regulatory mitochondrial proteins mitofusin-1, HSP60, and 14-3-3 protein theta are decreased in the *Mecp2^−/y^* neocortex. The expressional changes identified provide further details of the altered mitochondrial function and morphology in RTT. They emphasize brain-region-specific alterations of the mitochondrial proteome and support the notion of a metabolic component of this devastating disorder.

## 1. Introduction

Rett syndrome (RTT) is a complex neurodevelopmental disorder, which typically shows a delayed onset and affects mostly females [1,2]. Its primary cause is spontaneous mutations in the X-chromosomal *MECP2* gene, resulting in a loss of function of the encoded transcriptional modulator MeCP2 (methyl-CpG-binding protein 2) [3].

MeCP2 exerts a variety of modulatory functions, which include a negative control of transcription with gene silencing at certain developmental stages, whereas other genes are positively controlled [4]. In addition, the role of MeCP2 in alternative splicing has been confirmed [5], as well as direct interaction with histone proteins [6]. Accordingly, a general lack or limited function of MeCP2 culminates in the misregulation (either up or downregulation) of hundreds of other genes, which are pivotal for undisturbed cell functioning and metabolism.

Female patients affected by RTT seem to develop normally after birth since symptoms are barely obvious. The first clear signs of RTT, such as neurological and physical symptoms, start to develop after 7–18 months. The broad spectrum of clinical symptoms includes, among others, cardio-respiratory irregularities, motor dysfunction, severe cognitive impairment with a loss of already acquired speech, autism-like features, and an increased risk of epileptic seizures [1]. Microcephaly with reduced neuronal complexity are further typical features associated with RTT [7], and clear evidence for metabolic components of this disorder has been obtained [8,9]. This also involves mitochondria [10]. Male patients carrying *MECP2* mutations are quite rare; upon already developing severe symptoms shortly after birth, they usually do not survive their first year [11].

The very first notion of mitochondrial alterations in RTT patients was based on electron microscopical studies on the frontal lobe [12], as well as skeletal muscle [13], which confirmed enlarged, swollen, and translucent mitochondria presenting irregular cristae and granular inclusions. Whereas these early pioneer studies primarily focused on mitochondrial morphology, they were extended by reports on functional impairments of mitochondria. These included less negative mitochondrial membrane potentials and an increased ratio of FAD/NADH [14], increased respiration rates [15], an intensified oxidative burden [14,16], and lowered cerebral ATP content [17]. Furthermore, various pathways closely related to mitochondrial metabolism were found to be modulated in patient plasma [8] and MeCP2-deficient mouse neocortex [18]. Even changes in mitochondrial DNA were detected in patient blood samples [19].

In addition, variations in the expression and/or enzymatic activity of various proteins pivotal to mitochondrial function have been confirmed in RTT patients and mouse models. For example, reduced enzymatic activities were reported for succinate cytochrome c reductase (complex III) in skeletal muscle biopsies [13] and for cytochrome c oxidase (complex IV) in post-mortem brain tissue [20]. In MeCP2-deficient mouse brains, downregulation of NADH-dehydrogenase subunit 2 (complex I) and upregulation of cytochrome-b-c1 complex subunit 1 (complex III) were reported [15]. Furthermore, NADH:ubiquinone oxidoreductase (complex I), as well as ATP5B, a subunit of the FoF1 ATP synthase complex, were expressed at lower levels [21].

This variety of functional, structural, and genetic mitochondrial alterations unveiled in RTT patients and cell and mouse models of RTT is suspected to contribute to the impaired redox balance and the intensified oxidative burden associated with this disorder [22,23,24]. Intensified production of H_2_O_2_ in *Mecp2*-mutant mice seems to be accompanied by a modified activity of complex II [21,25]. Moreover, for RTT patient-derived dermal fibroblasts, increased cellular and mitochondrial oxidant production was reported [26]. Furthermore, patient blood samples showed decreased vitamin E serum levels [27] and diminished free-radical scavenging capacities [28]. Quantitative imaging of cellular redox conditions also confirmed an oxidative burden in the MeCP2-deficient mouse hippocampus [14,29].

An interesting aspect is that the mitochondrial alterations and the cerebral redox impairments are already detectable in neonatal MeCP2-deficient mice. Accordingly, these early markers precede the other characteristic symptoms of RTT and therefore may contribute to the further pathogenesis of this disorder [14,30]. The corresponding alterations and metabolic signatures seen in blood samples [8], dermal fibroblasts [26], and patient-derived neuronal progenitor cells [31] do support this concept.

As MeCP2-deficiency in particular affects brain development and gives rise to a range of neurological and cognitive symptoms, we focused here on the study of the mitochondrial proteome in brain tissue. In order to continue the deciphering of disease-related alterations in MeCP2-deficient brain tissue, we conducted our mitochondrial proteomic analyses in the neocortex and hippocampus of male symptomatic *Mecp2* knock-out *(Mecp2^−/y^)* mice. These brain regions were chosen, as they are pivotal for cognitive function. Furthermore, widespread morphological and ultrastructural changes have been reported earlier in dendritic widths, spine densities, and mitochondrial appearance in the motor cortex and hippocampus of MeCP2-deficient mice [32]. Various mitochondria-related proteins were expressed differentially in *Mecp2^−/y^* mice, including proteins crucially involved in mitochondrial respiration, mitochondrial metabolism, and mitochondrial organization. These identified expressional changes may contribute to explaining some of the morphological and functional mitochondrial alterations that are closely associated with the conditions of MeCP2-deficiency in hippocampal and neocortical tissue.

## 2. Materials and Methods

### 2.1. Animals and Brain Tissue Isolation

**Figure 1:**
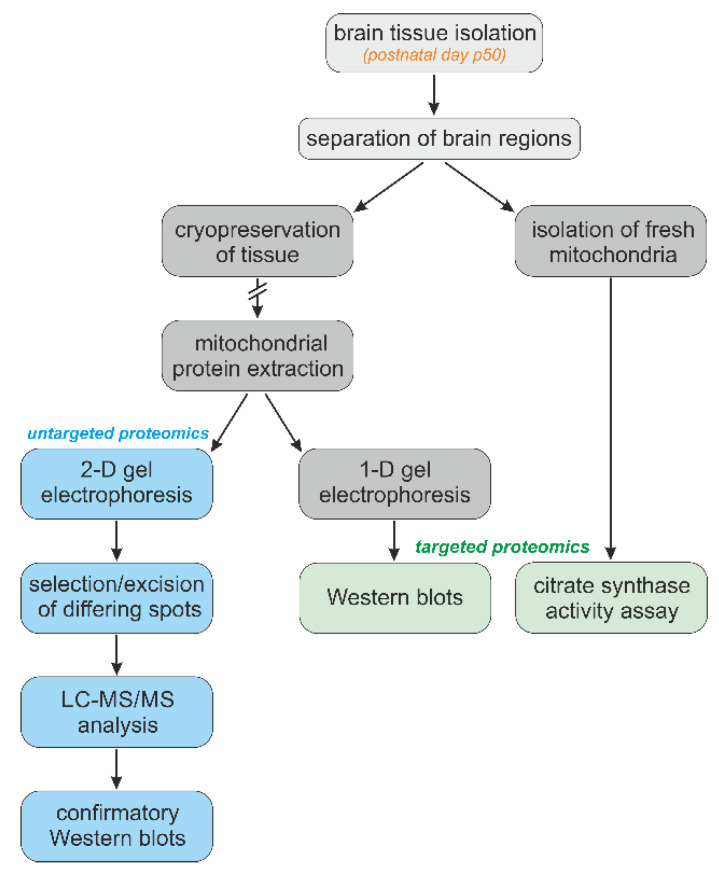
Experimental design including the sequence and hierarchy of the experimental procedures applied for the targeted and untargeted proteomic analyses of brain-derived mitochondria.

We continued using the *Mecp2* knockout mouse model of RTT [B6.129P2(C)-Mecp2tm1.1Bird] [33]. Even though RTT mostly affects girls, hemizygous male (*Mecp2^−/y^*) mice were used to guarantee clearly defined genetic conditions characterized by a total lack of MeCP2 in the studied tissues. We focused on the clearly symptomatic, severe disease stage that is typically present in *Mecp2^-/y^* mice around postnatal day p50. Mice were decapitated under deep anesthesia (diethylether). Their brains were rapidly isolated and immediately cooled down in ice-cold phosphate-buffered saline (PBS, 3–4 min). Then the different brain regions were carefully isolated and flash-frozen in liquid N_2_ and cryostored in the freezer (−80 °C) until further individual processing. The detailed sequence of the different experimental procedures is indicated as a graphical flowchart (Figure 1).

### 2.2. Isolation of Mitochondria

Mitochondrial fractions were obtained from frozen, cryopreserved tissue samples by a special mitochondrial isolation kit for tissue (89801, ThermoFisher Scientific, Waltham MA, USA) following the manufacturer’s instructions. It cannot be ruled out that the thawing of frozen tissue samples might have partly damaged mitochondrial membranes and that, as a result, some of the mitochondria were lost during the differential centrifugations. However, this would have been the case with all samples studied. Therefore, a genotype-dependent bias is more than unlikely. To be able to treat the mitochondrial pellet separately for 2-D and 1-D electrophoresis, the samples were divided into two parts before the last centrifugation. For 1-D electrophoresis and subsequent Western immunoblotting, the pellet was dissolved in a 5% sodium dodecylsulphate (SDS) solution, whereas for 2-D electrophoresis, pellets were dissolved in a buffer composed of 7 M urea, 2 M thiourea, 2% CHAPS, 2% pharmalyte 3–10, 2% Triton X-100, 0.1% ASB-14,2-mercaptoethanol, and bromophenol blue.

The mitochondria-enriched suspensions required for the citrate-synthase activity (CSA) assay were prepared from freshly isolated distinct brain regions by manual grinding in a micro-potter, according to our previously published procedures of differential centrifugation [21].

### 2.3. Protein Determination

Protein concentrations of the individual samples were quantified (four technical repeats) by a micro bicinchoninic acid protein assay (23235, Pierce/ThermoFisher Scientific). Alternatively, a Bradford-type assay (Protein Assay #5000006, BioRad, Hercules CA, USA) served to quantify the total protein content of the mitochondrial suspensions isolated from fresh brain tissue for the CSA assay.

### 2.4. Citrate-Synthase Activity Assay

The CSA assay was conducted on isolated mitochondria, according to the laboratory protocol provided by Oroboros Instruments (Innsbruck, Austria) (https://wiki.oroboros.at/index.php/MiPNet17.04_CitrateSynthase) (accessed on 10 August 2021). As this assay represents an optical spectrometer-based colorimetric test, it could not be conducted reasonably on the crude brain tissue homogenates. The large amounts of tissue debris would have prevented any spectrophotometric optical detection. This is why mitochondria-enriched suspensions of the respective brain regions were used here for the quantification of CSA. There will be some loss of mitochondria during the differential centrifugation, but this loss can be expected to be very similar among brain regions and genotypes. Stock solutions of Tris-HCl buffer (1.0 M, pH 8.1), Tris-HCl buffer (0.1 M, pH 7.0), and triethanolamine-HCl buffer (0.5 M, pH 8.0), also containing EDTA (5 mM) and Triton X-100 (10%), were prepared monthly. Before each experiment, we freshly dissolved 6.6 mg oxaloacetate in 5 mL of triethanolamine-HCl-buffer (0.1 M, pH 8.0). Moreover, 2 mg DTNB was freshly dissolved in a 5 mL volume of Tris-HCl-buffer (pH 8.1).

A spectrophotometer (flx-Xenius, SAFAS, Monaco-Ville, Monaco) was used with a duration setting of 3 min, an absorption wavelength of 412 nm, and a sampling interval of 10 s. The cuvette temperature was set to 35 °C. Quartz cuvettes contained the sample volume (Vsample, with 30 µg protein) filled up with dH_2_O to 800 µL, also adding 25 µL of 10% Triton X-100, 25 µL of an aqueous 12.2 mM acetyl CoA stock solution, and 100 µL of the 1.01 mM DTNB stock. Samples were first measured for 3 min to obtain the baseline. Afterwards, 50 µL of oxaloacetate solution was added to each cuvette, and CSA activity was measured for 3 min. Each brain region (CTX; HPC; MB; CB; BS) was measured twice, and the respective data were averaged per mouse. The first 3 min of measurement (baseline recording) was subtracted from the subsequent CSA measurements, and the specific enzyme activity (ν) was calculated by the following equation:

$$v=\frac{\mathrm{rA}}{l\;*\;\varepsilon_{\mathrm{TNB}}{\;*\;v}_{\mathrm{TNB}}}\;*\;\frac{\mathrm{Vcuvette}}{\mathrm{Vsample}\;*\;\rho }\;$$

In detail, rA stands for the rate of absorbance change; l represents the length of the optical path (1 cm); ε_TNB_ is the extinction coefficient of TNB; ν_TNB_ is the stoichiometric number of TNB (which is 1); Vcuvette represents the volume of solution in the cuvette; Vsample is the volume of added sample, and ρ the mass concentration of the added sample.

### 2.5. 1-D Electrophoresis and Western Blotting

Aliquots of 30 µL of buffer (1 µg/µL protein content) were used for SDS gel electrophoresis and Western blotting [34]. Proteins were transferred from polyacrylamide gels onto nitrocellulose membranes and membranes were stained with the Ponceau S solution 0.1% (*w*/*v*) to confirm successful transfer and uniform gel loading and were next digitized and analyzed using the ImageJ software (https://imagej.net/software/imagej) (accessed on 22 September 2021). After washing with PBS + 0.05% Tween 20, the standard immunochemical staining was conducted with peroxidase-labeled secondary antibodies and the Super-Signal West Pico Chemiluminescent substrate (Pierce, 34580). To achieve the linearity of response, the nitrocellulose membranes were then exposed with intensifying screens to X-ray films, which had been pre-flashed with Sensitize (RPN2051, Amersham, Amersham, UK). The following antibodies were used: (1) For the quantitation of components of the oxidative phosphorylation system (OXPHOS), Total OXPHOS rodent WB antibody cocktail (ab110413, Abcam, Cambridge, UK); (2) for DRP, ab184247 (Abcam); (3) for mitofusin-1, ab57602 (Abcam); (4) for protein 14-3-33, sc-59414 (Santa-Cruz Biotechnology, Dallas, TX, USA); (5) for creatine kinase type B, ab125114 (Abcam); and (6) for HSP 60, sc-13115 (Santa-Cruz Biotechnology).

The optical density of the respective protein bands was measured using LabWorks 4.0 (UVP). For the calibration of all autoradiographs, we included four internal protein standards in each of the gels. These standards comprise the mitochondrial fraction isolated from the WT mouse brains. The same standards were used in all of the experiments conducted, and they contained 15, 30, 45, and 60 μg of total protein, respectively. Optical densities were directly proportional to the amount of loaded proteins. The respective measures (Figures 4, 5, and 7–9) were obtained by dividing the individual optical band densities of a given sample (e.g., *Mecp2^-/y^* neocortex) by the optical density, which, from the calibration of the very autoradiograph, corresponded to 30 μg of total protein in the standard. The data obtained by this normalization are indicated as the ”relative amount” of, e.g., mitofusin-1. As only 20 wells were available on the gels, the four standards were loaded as a single copy.

These densities of the respective bands were not referred (normalized) to any mitochondrial or other cellular housekeeping proteins, such as actin, since it cannot be taken for granted that such reference proteins remain unchanged under our experimental conditions. Therefore, to rule out any unreliability that might arise from normalization to housekeeping proteins [35], we consistently verified proper sample loading by Ponceau S staining as well as by the calibration with the mentioned protein standards (see also [34]).

### 2.6. 2-D Electrophoresis

Before isoelectric focusing was performed, the strips (linear pH 3.0–10.0, 18 cm) first had to be rehydrated by overnight incubation in a buffer containing 8 M urea, 0.5% pharmalyte 3–10, 0.5% Triton X-100, and a 0.1% DeStreak reagent. Isoelectric focusing was then performed at 500 V for 3 h and at 3500 V for 18 h. Each strip was loaded with 40 µg of the mitochondrial protein. After isoelectric focusing, strips were equilibrated first for 15 min in a buffer composed of 0.05 M Tris-HCl (pH 6.8), 6 M urea, 30% glycerol, 3% SDS, and 1% DTT and then for another 15 min in a similar buffer, in which DTT was replaced by 2.5% iodoacetamide. For SDS electrophoresis, we used 12.5% polyacrylamide gels of 1 mm thickness. The temperature was 25 °C, and two sequential voltage settings were applied (1–10 mA/gel, 80 V for 1 h, followed by 12 mA/gel, 150 V for 18 h).

### 2.7. Staining, Scanning, and Image Processing

The gels underwent silver staining (kit, GE Healthcare, Chicago, IL, USA); the glutaraldehyde step was omitted. The silver-stained gels were then imaged with a Labscan6.0 Image Scanner III (GE Healthcare), digitized, and further processed with ImageMaster 2-D platinum 7.0 software. This software does not calculate spot intensities, as this parameter might be subject to certain variations among gels, but rather compares protein amounts by their relative amounts in the loaded samples (expressed here as %Volume). This %Vol parameter represents the relative volume of a given spot. It is a normalized value that remains relatively independent of variations due to protein loading and staining by considering the total volume over all spots in an image.

For the best comparability of experimental conditions, six gels (three *Mecp2^−/y^* and three WT brain samples from the neocortex and hippocampus) were run in parallel on a 2-D electrophoresis system (GE Healthcare). In total, 6 tissue samples of the hippocampus and neocortex were analyzed for each genotype. The data of those spots coinciding with the location (pI and molecular weight) and revealing a differential expression by at least 2-fold in the different experiments were assessed by two-tailed Student’s *t*-tests for potential genotype-related differences. A significance level of 5% was chosen. Those spots that were significantly differentially expressed were excised and stored at less than –20 °C until further MS analysis.

### 2.8. In-Gel Digestion and Subsequent MS Analysis

The excised spots were destained with 30 mM potassium ferricyanide and 100 mM sodium thiosulfate. Gel pieces were then washed and incubated with 0.1 M sodium hydrogencarbonate and washed again. The gels were reduced by dithiothreitol (DTT) and alkylated by iodoacetamide followed by trypsin (Thermo Fisher MS grade, 90057) overnight digestion at 37 °C. The next day, the extracted peptides were transferred into a sample vial and loaded into an autosampler for automated LC-MS/MS analysis.

Mass spectrometry analysis was conducted using LCQ FLEET Ion Trap with the nano HPLC system Easy nLC-1000 (ThermoFisher Scientific, Waltham, MA, USA). Peptide separation was achieved by reverse-phase chromatography, and the MS/MS spectra obtained were analyzed using the SEQUEST search algorithm (Proteome Discoverer 1.4, ThermoFisher Scientific). The SEQUEST search parameters were set for default parameters. Only those unique peptide matches that passed the filter and also met the strict false discovery rate (FDR) criteria (*p* ≤ 0.01) were considered. Such peptide data were then searched against the UniProt UniRef100 *Mus Musculus* species protein databases. A summary of further details on the identified proteins is provided in Supplementary Table S1. It should be noted that our MS system, which is equipped with an ion trap analyzer, is not meant to provide quantitative data on the protein levels included in the differentially appearing spots but rather provide qualitative information. Accordingly, the MS analyses performed served to identify these proteins being present but cannot be used as an independent confirmation of the extent of differential protein expression determined by the 2-D gels. With the strict FDR values applied, it was very rare to identify two or even more proteins in an excised gel spot. In a few cases (see Supplementary Table S1), the identified proteins had largely different molecular weights, which allowed us to reject the wrong possibility.

### 2.9. Statistical Analysis

This study was conducted on brain tissue isolated from a cohort of 14 male wildtype and 14 *Mecp2^−/y^* mice (postnatal day 50). The data for the hippocampus and neocortex were analyzed separately. As experiments involved only two groups of mice, an unpaired Student’s *t*-test was applied, unless stated differently. All statistical tests conducted were two-tailed, and all significant differences reaching a significance level of at least *p* < 0.05 were reported. For multiple comparisons (the CSA assay), a one-way ANOVA followed by Dunnett´s post-hoc test was used. Throughout the manuscript, data are reported as mean ± standard deviation; the number of observations (n) is the number of mice analyzed.

## 3. Results

### 3.1. Citrate Synthase Activities Differ among Brain Regions

**Figure 2:**
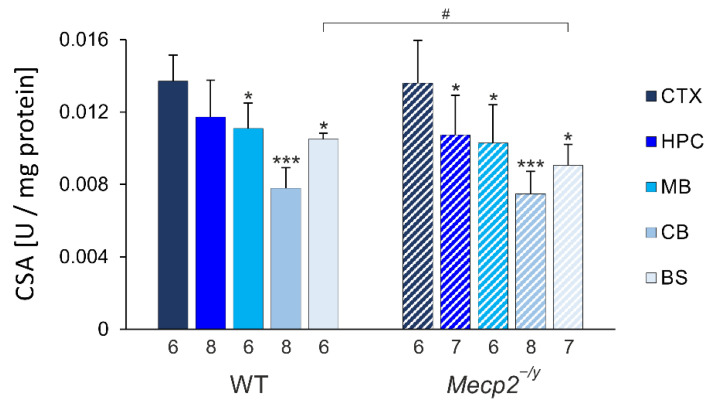
Quantification of citrate-synthase activity (CSA) defines clear brain-regional differences in WT and *Mecp2^−/y^* mice. With neocortex (CTX) showing highest and cerebellum (CB) showing lowest activities, hippocampus (HPC), midbrain (MB), and brainstem (BS) present intermediate levels. RTT-related changes in CSA were uniquely found in brainstem. Data are plotted as means ± standard deviation. Genotype-matched brain regional differences as compared to neocortex are identified by asterisks (* *p* < 0.05, *** *p* < 0.001; one-way ANOVA mixed-effect analysis, post-hoc Dunnett´s multiple comparison test). RTT-related differences among genotypes are identified by crosshatches (# *p* < 0.05; unpaired 2-tailed Student´s *t*-test). The number of mice studied is reported below each of the bars plotted.

In view of the functional mitochondrial alterations associated with RTT, one might expect a compensatory increase in mitochondrial mass. We, therefore, conducted a spectrophotometric citrate synthase activity (CSA) assay as an estimate of mitochondrial contents within the different brain regions of male wildtype (WT) and *Mecp2^−/y^* mice. These analyses revealed very distinct regional differences (Figure 2).

The highest citrate synthase activities were consistently detected in the neocortex (CTX), whereas the lowest activity was always found in the cerebellum (CB). The hippocampus (HPC), midbrain (MB), and brainstem (BS) showed intermediate levels. The corresponding order of activities was also found in *Mecp2^−/y^* mice. The genotypic comparison of citrate synthase activities among WT and *Mecp2^−/y^* mice hardly revealed any obvious differences. Only in *Mecp2^−/y^* brainstems was significantly lower citrate synthase activity confirmed as compared to WT mice.

### 3.2. The OXPHOS System Appears Largely Unaffected in a First Targeted Screening

In the initial steps of our experiments, we inquired, using a targeted screening approach, if there are any differences in the relative expression levels of respiratory chain complexes I, II, III, and IV or the ATP synthase FoF1 complex in the hippocampus and neocortex of *Mecp2^−/y^* and WT mice. The following proteins were quantitatively evaluated by 1-D electrophoresis/Western immunoblotting: (1) NADH: ubiquinone oxidoreductase subunit B8 (NDUFB8), complex I; (2) succinate dehydrogenase complex iron sulfur subunit B (SDHB), complex II; (3) ubiquinol-cytochrome C reductase core protein 2 (UQCRC2, alternate name cytochrome B-C1 complex subunit 2), complex III; (4) cytochrome c oxidase subunit 1 (MTCO1), complex IV; and (5) ATP synthase F1 subunit alpha (ATP5A), FoF1 ATP synthase complex. These targeted proteins represent complex subunits that are labile when their complex is not assembled. Accordingly, detecting these subunits does provide detailed information about the relative levels of the individual complexes (see: https://www.abcam.com/total-oxphos-rodent-wb-antibody-cocktail-ab110413.html) (accessed on 5 December 2022).

**Figure 3:**
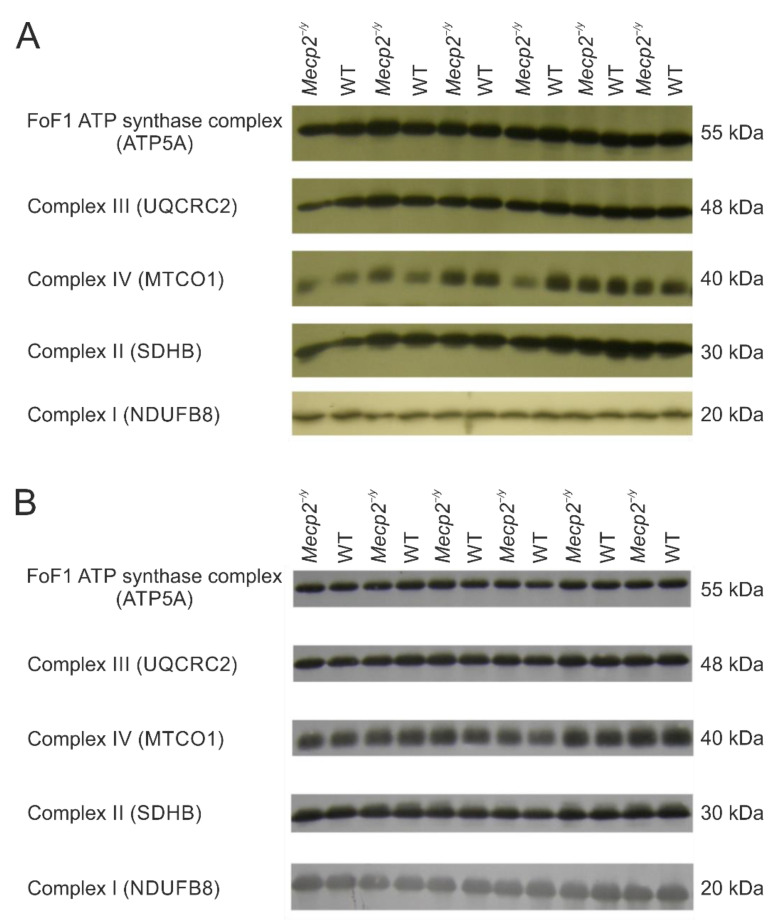
Expression of selected representative subunits of the mitochondrial OXPHOS system in hippocampus (**A**) and neocortex (**B**) of *Mecp2^−/y^* and WT mice. Lanes were alternatingly loaded with *Mecp2^−/y^* and WT samples. No significant genotype-related differences were detected for the chosen subunits of the various respiratory chain complexes or the FoF1 ATP synthase complex (see also Table 1). See Supplementary Figure S1 for the original Western blot images.

No genotype-related significant differences were observed for the studied proteins—neither in the hippocampus nor in the neocortex (Figure 3, Table 1). However, relative levels of components of the OXPHOS system do not provide exclusive information about complex composition, which could be subjected to a variety of changes. These data also do not exclude the possibility of other differences in the whole mitochondrial proteome between WT and *Mecp2^−/y^* mice.

### 3.3. Regulators of Mitochondrial Fusion/Fission Dynamics Are Partly Decreased in RTT Mice

**Figure 4:**
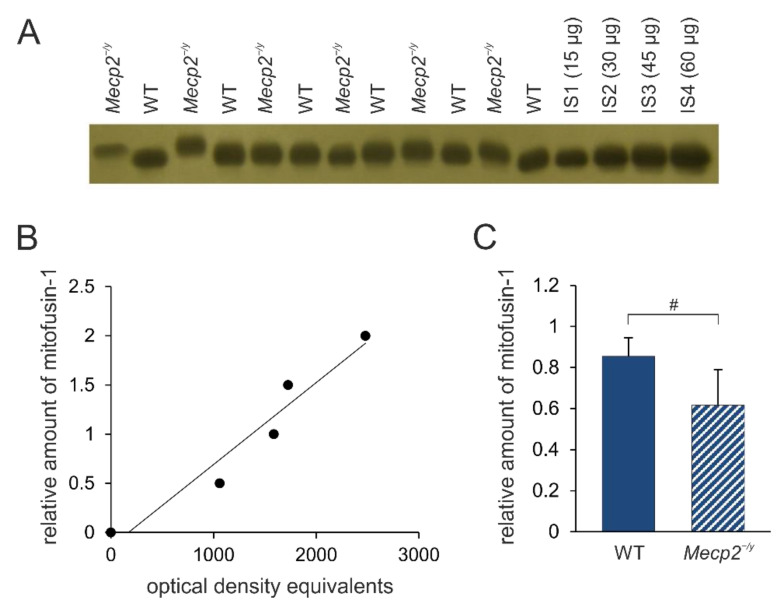
Mitofusin-1 expression in the neocortex of *Mecp2^−/y^* and WT mice. (**A**) Sample film; each lane contains an individual sample. IS1-IS4 represent internal standards containing 15, 30, 45, and 60 μg of protein, respectively. (**B**) The calibration plot was fitted by a linear least-squares regression. (**C**) Mean expression levels of mitofusin-1 (mean ± standard deviation) in WT and *Mecp2^−/y^* neocortex. RTT-related differences among genotypes are identified by crosshatches (# *p* < 0.05; unpaired 2-tailed Student´s *t*-test). See Supplementary Figure S1 for the original Western blot images.

Changes in mitochondrial morphology seem closely associated with RTT. Therefore, we next assessed the expression levels of proteins that are known to be crucially involved in the regulation of mitochondrial fusion/fission dynamics (e.g., mitofusin-1, Mit-1; dynamin-related protein 1, DRP-1). The amount of mitofusin-1 is significantly less in the neocortex of *Mecp2^−/y^* mice compared to WT mice (*t*-value = 2.47, *p*-value = 0.033, df = 10) (Figure 4). No significant differences were found for DRP-1 in the neocortex (WT: 0.832 ± 0.187, *Mecp2^−/y^*: 0.734 ± 0.339, n = 6 each; *p* = 0.549).

**Figure 5:**
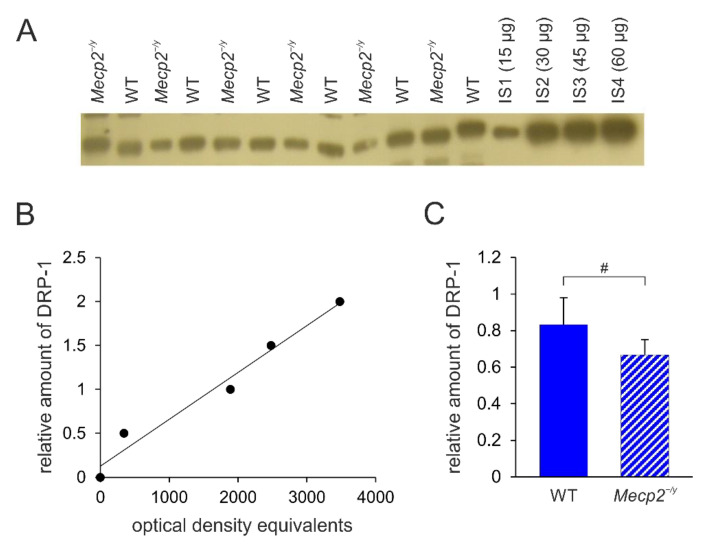
DRP-1 expression in the hippocampus of Mecp2^−/y^ and WT mice. (**A**) Sample film; each lane contains an individual sample. Lanes IS1-IS4 represent the internal standards containing 15, 30, 45, and 60 μg of protein, respectively. (**B**) The calibration plot was fitted by a linear least-squares regression. (**C**) Mean levels of DRP-1 (mean ± standard deviation) in the hippocampus of the two different groups of mice. RTT-related differences among genotypes are identified by crosshatches (# *p* < 0.05; unpaired 2-tailed Student´s t-test). See Supplementary Figure S1 for the original Western blot images.

In the *Mecp2^−/y^* hippocampus, significantly less DRP-1 was detected compared to WT mice (*t* = 2.39, *p* = 0.038, df = 10) (Figure 5). The amount of mitofusin-1 in the hippocampus did not differ significantly among the two genotypes (WT: 0.43 ± 0.06, *Mecp2^−/y^*: 0.34 ± 0.15, n = 5 each, *p* = 0.234).

### 3.4. The Mitochondrial Proteome Clearly Differs among Mecp2^-/y^ and WT Mice

**Figure 6:**
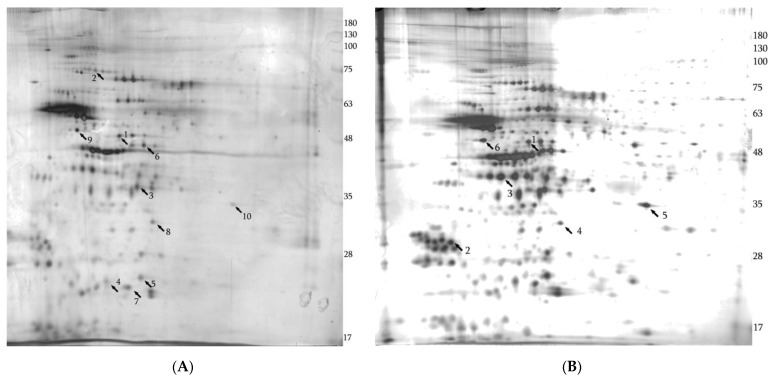
Silver-stained 2-D electrophoresis gels of the mitochondrial fractions isolated from *Mecp2^−/y^* hippocampus and neocortex. The arrows indicate significantly changed proteins identified by MS. Arrows in the brackets indicate the direction of changes compared to WT mice. (**A**) Hippocampus: 1—cytochrome b-c1 complex subunit 1, mitochondrial (↑); 2—NADH-ubiquinone oxidoreductase 75 kDa subunit, mitochondrial (↑); 3—pyruvate dehydrogenase E1 component subunit beta, mitochondrial (↑); 4—NADH dehydrogenase [ubiquinone] iron-sulfur protein 8, mitochondrial (↑); 5—NADH dehydrogenase [ubiquinone] flavoprotein 2, mitochondrial (↑); 6—creatine kinase B-type (↑); 7—ATP synthase subunit d (↑); 8—prohibitin 1 (↑); 9—gamma-enolase (↓); 10—cAMP-dependent protein kinase catalytic subunit alpha (↓). (**B**) Neocortex: 1—cytochrome b-c1 complex subunit 1, mitochondrial (↑); 2 -14-3-3 protein theta (↓); 3—guanine nucleotide-binding protein G(o) subunit alpha (↑); 4—prohibitin 1 (↑); 5—cAMP-dependent protein kinase catalytic subunit alpha (↓); 6—gamma enolase (↓). See Supplementary Figure S2 for the full collection of 2-D gel images.

To obtain a broader overview of the differently regulated mitochondrial proteins in *Mecp2^−/y^* brain tissue, we took advantage of 2-D electrophoresis. Indeed, 2-D electrophoresis of mitochondrial fractions isolated from the hippocampus and neocortex of *Mecp2^−/y^* and WT mice revealed several protein bands with statistically significant differences, both upregulated and downregulated. Representative images of two silver-stained 2-D gels are displayed in Figure 6; the complete collection of 2-D gels is included in the Supplementary Figure S2 (“Silver-stained 2-D gels”). The identities of these bands were classified further by subsequent MS analysis (Table 2). Details for the MS analysis of differentially expressed proteins are provided in Supplementary Table S1 (“MS Data”). In sum, 10 differentially expressed proteins were identified in the hippocampus and 6 differentially expressed proteins were found in the neocortex. From these differentially expressed proteins, four show corresponding changes in both brain regions: Cytochrome b-c1 complex subunit 1 and prohibitin 1 are upregulated in the hippocampus and neocortex of *Mecp2^−/y^* mice, whereas gamma-enolase and cAMP-dependent protein kinase catalytic subunit alpha are downregulated in the hippocampus and neocortex of *Mecp2^−/y^* mice compared to WT conditions.

### 3.5. Confirmatory Western Blot Analyses of Selected Differentially Expressed Proteins

To verify the results from 2-D electrophoresis, we performed Western immunoblotting experiments on two selected proteins. One candidate was chosen from the differentially expressed proteins in the hippocampus and one was chosen from the neocortex. Furthermore, the selection of proteins was based on (1) the regional specificity as the protein should be differentially expressed in only one of the studied regions, i.e., either the hippocampus or the neocortex, and (2) the availability of reliable commercial antibodies. Based on these parameters, we selected creatine kinase B and 14-3-3 protein theta.

**Figure 7:**
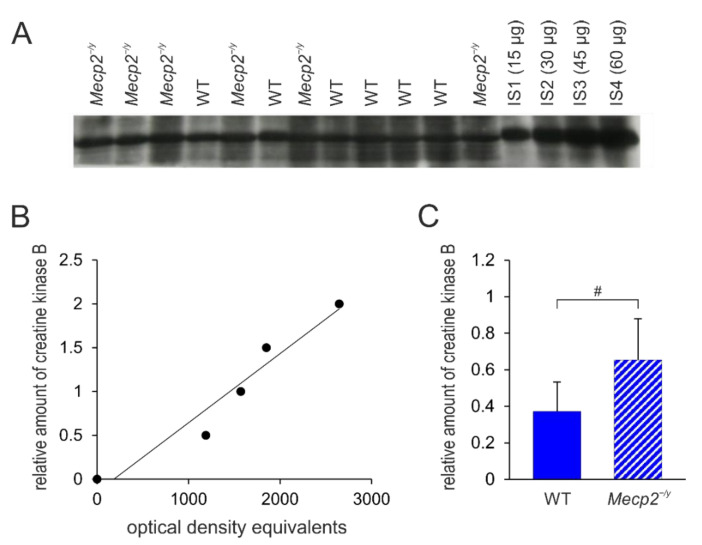
Creatine kinase B protein expression levels in the hippocampus of *Mecp2^−/y^* and WT mice. (**A**) Sample film; each lane contains an individual sample. Lanes IS1-IS4 show internal standards containing 15, 30, 45, and 60 μg of protein, respectively. (**B**) The calibration plot was fitted by a linear least-squares regression. (**C**) Statistical comparison of the mean levels of creatine kinase B (mean ± standard deviation) in the hippocampus of the two groups of mice. RTT-related differences among genotypes are identified by crosshatches (# *p* < 0.05; unpaired 2-tailed Student´s *t*-test). See Supplementary Figure S1 for the original Western blot images.

According to our 2-D analysis, creatine kinase B is upregulated in the hippocampus of *Mecp2^−/y^* mice but is not differentially expressed in the *Mecp2^−/y^* neocortex (see Table 2). In confirmatory Western blots, antibodies against creatine kinase B reacted with a 50 kDa protein (Figure 7A), and the mean amount of protein was confirmed to be significantly higher in the hippocampus of *Mecp2^−/y^* mice compared to WT mice (*t* = 2.41, *p* = 0.037, df = 10, Figure 7C). As expected, the amount of creatine kinase B in the neocortex did not differ among genotypes (WT: 0.77 ± 0.21, *Mecp2^−/y^*: 0.63 ± 0.19, n = 6 each, *p* = 0.240).

**Figure 8:**
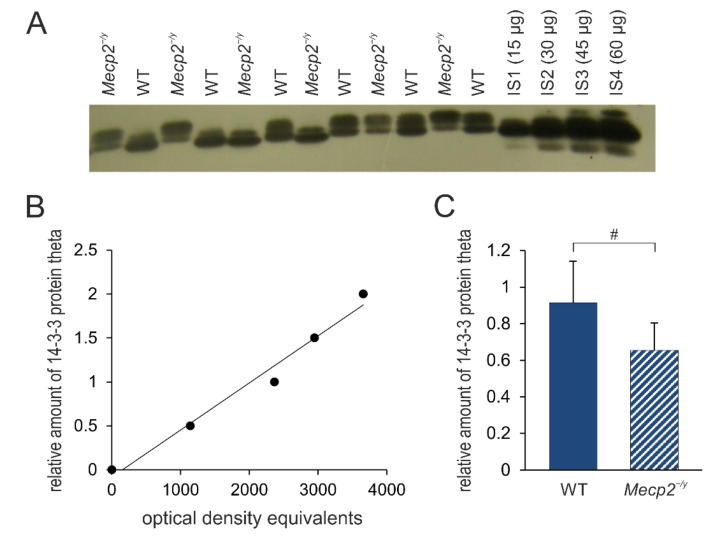
Summary of 14-3-3 protein theta expression levels in the neocortex of *Mecp2^−/y^* and WT mice. (**A**) Sample film; each lane contains an individual sample. Lanes IS1-IS4 are internal standards containing 15, 30, 45, and 60 μg of protein, respectively. (**B**) The calibration plot was fitted by a linear least-squares regression. (**C**) Mean levels of 14-3-3 protein theta (mean ± standard deviation) in the hippocampus of the two groups of mice. RTT-related differences among genotypes are identified by crosshatches (# *p* < 0.05; unpaired 2-tailed Student´s *t*-test). See Supplementary Figure S1 for the original Western blot images.

Our 2-D electrophoresis also suggests that the neocortex of *Mecp2^−/y^* mice expresses less 14-3-3 protein theta (see Table-1), and Western immunoblotting analysis validated this difference (Figure 8). The antibody against this protein reacted with a band of 26 kDa molecular weight corresponding to 14-3-3 protein theta, and the mean expression of this protein was significantly less (~ 45%) in *Mecp2^−/y^* neocortex compared to WT (*t* = 2.35, *p* = 0.04, df = 10). In the hippocampus, only a trend, rather than a significant reduction, was detected for 14-3-3 protein theta (WT: 1.14 ± 0.45, *Mecp2^−/y^*: 0.80 ± 0.18, n = 6 each, *p* = 0.114).

**Figure 9:**
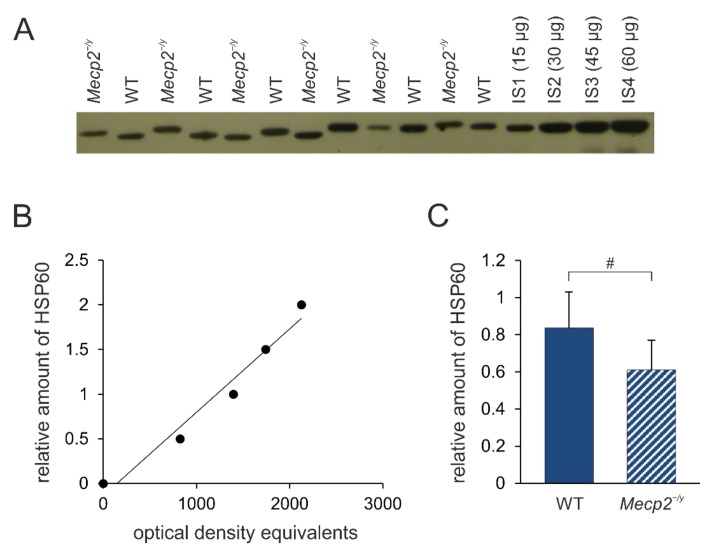
HSP60 expression levels in the neocortex of *Mecp2^−/y^* and WT mice. (**A**) Sample film; each lane contains an individual sample. Lanes IS1-IS4 are internal standards containing 15, 30, 45, and 60 μg of protein, respectively. (**B**) The calibration plot is fitted by a linear least-squares regression. (**C**) Mean levels of HSP60 (mean ± standard deviation) in the neocortex of the two groups of mice. RTT-related differences among genotypes are identified by crosshatches (# *p* < 0.05; unpaired 2-tailed Student´s *t*-test). See Supplementary Figure S1 for the original Western blot images.

Heat shock protein 60 (HSP60), which is pivotal for mitochondrial protein homeostasis, is an interacting partner of 14-3-3 protein [36] and is involved in neurodegeneration [37]. Therefore, as the next step, we inquired whether the expression levels of HSP60 are affected in the neocortex and hippocampus of *Mecp2^−/y^* and WT mice. The level of HSP60 in the *Mecp2^−/y^* neocortex is significantly less compared to WT mice (*t* = 2.24, *p* = 0.049, df = 10, Figure 9), whereas for the hippocampus, no significant differences were observed (WT: 0.786 ± 0.225, *Mecp2^−/y^*: 0.636 ± 0.250, n= 6 each, *p* = 0.3).

## 4. Discussion

Our findings convincingly indicate distinctive alterations in the neocortical and hippocampal mitochondrial proteomes of *Mecp2^−/y^* and WT mice. These data were obtained by an ad hoc approach, i.e., by choosing proteins involved in mitochondrial dynamics, as well as quantifying brain regional citrate synthase activities as a marker of mitochondrial mass. In addition, we conducted analyses, which do not make any assumptions about particular proteins, by performing the proteomic approach of 2-D electrophoresis and subsequent MS analysis. The observed differences were further validated for two selected proteins by Western immunoblotting. Interestingly, all of the differentially expressed mitochondrial proteins identified by us are nuclear-encoded proteins.

We found bidirectional changes in the expression of mitochondrial proteins in the neocortex and hippocampus of *Mecp2^−/y^* mice. These results can be linked to the well-accepted view that MeCP2 functions not only as a negative regulator (silencer) of gene transcription but in some genes also activates transcription [4]. Accordingly, loss-of-function mutations in the *MECP2* gene give rise to an up-regulation of some genes but a downregulation of others, which was seen in the current study. We do not rule out also the possibility that some of these differences are secondary to MeCP2 deficiency.

### 4.1. Brain-Regional Differences in Mitochondrial Mass

By quantifying citrate synthase activity, obvious genotype-matched differences among the various brain regions were obtained for WT and *Mecp2^−/y^* mice. Mitochondrial contents were always highest in the neocortex, somewhat lower in the hippocampus, midbrain, and brainstem, and consistently lowest in the cerebellum. The extents of these brain-regional differences were comparable for WT and *Mecp2^−/y^* mice, and they can be assumed to reflect the specific metabolic demand of the respective brain regions. Genotype-related, i.e., RTT-associated, differences between WT and *Mecp2^−/y^* mice were uniquely confirmed in the brainstem. The *Mecp2^−/y^* brainstem contained significantly fewer mitochondria than the WT brainstem. This finding may be of interest in view of the severe irregularities in cardiorespiratory control, which are among the hallmarks of RTT [38]. The indifferent mitochondrial contents of the *Mecp2^−/y^* hippocampus and the *Mecp2^−/y^* neocortex in comparison to WT mice are in line with our earlier findings when we compared the relative protein expression levels of the mitochondrial marker VDAC3 (normalized to GAPDH) in tissue homogenates of the hippocampus and neocortex of adult male mice [21].

### 4.2. Mitochondrial Fusion/Fission Dynamics Are Impaired in RTT

RTT patients and mouse models of this disorder are characterized by abnormal mitochondrial morphology and sizes [12,13]. Mitochondria are more numerous and their total mass is increased in cultured neonatal *Mecp2^−/y^* astrocytes compared to WT conditions [39]. Therefore, altered mitochondrial fusion and fission processes (mitochondrial dynamics) could be involved in RTT pathology. Our data revealed a decreased amount of mitofusin-1 in the neocortex but not in the hippocampus of *Mecp2^−/y^* mice. Decreased mitochondrial fusion can at least partially contribute to the increased number of mitochondria and their altered morphology in RTT. We suggest that such defects in mitochondrial fusion/fission dynamics may also augment abnormal mitochondrial functioning.

It is well documented that various CNS diseases are characterized by disturbed mitochondrial dynamics (reviewed in [40]). Aberrant mitochondrial fusion/fission processes in astrocytes are closely linked to their inflammatory activation [41]. Furthermore, as confirmed in preclinical studies, reestablishing mitochondrial homeostasis by intensified mitochondrial biogenesis opposes injury progression and increases functional recovery [40].

In contrast to impaired mitochondrial dynamics, learning and memory are associated with increased amounts of mitofusin-1 and DRP-1, and the actual levels of these proteins correlate with learning efficiency [42]. As *Mecp2*-mutant mice show profound learning and memory impairments [43], a decreased amount of mitofusin-1 might contribute to these very aspects of the complex pathological conditions associated with this disorder.

On the 2-D gels, mitofusin-1 was not detected as a differentially expressed protein. However, only those proteins that showed at least 2-fold expression differences on the gels were selected for subsequent MS analysis. Therefore, mitofusin-1 might not have met these selection criteria.

In RTT patient-derived skin fibroblasts, impaired quality control of mitochondria was reported to give rise to obvious morphological alterations, such as hyper-fused mitochondria [44]. As a mechanistic background, an upregulation of mitofusin-1 and mitofusion-2 was confirmed, whereas dynamin-related protein 1 and mitochondrial fission 1 protein were downregulated. Furthermore, PINK1/Parkin-mediated mitochondrial removal was impaired in these fibroblasts [44].

### 4.3. Complex I Components of the OXPHOS System

Complex I represents the biggest and most complicated enzyme complex within the mitochondrial respiratory chain [45]. Three members of this complex, namely the NADH-ubiquinone oxidoreductase 75 kDa subunit (Ndufs1), NADH dehydrogenase [ubiquinone] iron-sulfur protein 8 (NDUFS8), and NADH dehydrogenase [ubiquinone] flavoprotein 2 (NDUFV2), were upregulated in the *Mecp2^−/y^* hippocampus compared to WT conditions. However, the assessment of the relative level of complex I, by the quantification of NADH:ubiquinone oxidoreductase subunit B8 (NDUFB8) in the hippocampus and neocortex, did not reveal any genotype-related differences among the studied mouse strains (Table 1). The situation is analogous to the cytochrome b-c1 complex subunit 1 differences (see below).

Complex I activity is not different between the rat brain neocortex of 6- and 15-month-old rats [46]. Nevertheless, 2-D electrophoresis analysis revealed a reduction of NDUFV2 and NDUFS1 subunits in 15-month-old rats [46]. The NDUFV2 protein contains a binuclear Fe–S cluster, potentially functioning as an antioxidant electron carrier, but it does not participate in the electron transfer from FMN to ubiquinone [45]. Taking into account the oxidative burden associated with RTT, this upregulation could serve as a compensatory mechanism. In any case, the changes observed for the selected complex I subunits point towards the plasticity of this complex in mitochondrial- and cellular functioning, and they may represent possible compensatory responses under pathological conditions of RTT.

Lower protein levels of complex I subunits encoding genes NDUFV2 and NDUFS1 were identified in various brain regions of patients affected by Down syndrome or Morbus Alzheimer’s [47] and schizophrenia [48]. In a severely affected RTT patient, reduced NADH dehydrogenase enzyme activity was detected [49]. Moreover, in various *Mecp2*-mutant mouse models, potential changes in complex I composition and/or activity have been identified. Female *Mecp2*-mutant mice show reduced protein expression levels of core complex I (NDUFS8), as well as complex II subunits (SDHB sub) [25]. Earlier, we also detected a slightly reduced level of a distinct complex I core subunit (NDUFB8) in the *Mecp2^−/y^* hippocampus and neocortex compared to WT conditions [21]. It should be noted, however, that in our earlier study, GAPDH expression served as a reference, whereas an internal protein standard was used here. On the transcript level, reduced mRNA levels of NADH dehydrogenase subunit 2 were detected in mitochondrial preparations extracted from the full brains of late-symptomatic, but not in those of early symptomatic, MeCP2-deficient mice [15].

### 4.4. Complex III Components of the OXPHOS System

Based on our 2-D-gel analyses, both the *Mecp2^−/y^* hippocampus and the *Mecp2^−/y^* neocortex show an increased expression of cytochrome b-c1 complex subunit 1. This protein is part of the multi-subunit transmembrane complex ubiquinol-cytochrome c oxidoreductase, which represents complex III within the mitochondrial electron transfer pathway. Interestingly, in support of our findings and the potential role of cytochrome b-c1 complex subunit 1 in RTT pathology, an earlier differential display approach revealed only one gene, which is upregulated in mitochondria extracted from the full brains of *Mecp2^−/y^* mice, namely *UQCRC1* [15]. In detail, a ~1.7-fold upregulation of UQCRC1 was found at the transcript level (mRNA) in full brain mitochondria at early and late symptomatic disease stages. Any corresponding alterations in protein expression, however, could not be detected by Western blots [15]. Our Western blot analysis of OXPHOS components also included the evaluation of the cytochrome b-c1 complex subunit 2 (Table 1), but no significant differences were found in either the hippocampus or the neocortex for that subunit. Along this line, we were not able to find any published data on these two subunits being affected identically by various treatments or under pathological conditions (see below).

Cytochrome b-c1 complex subunit 1 was found to be upregulated in the hippocampus of mice chronically treated with nicotine [50]. It is necessary to note that this was detected in the whole cellular proteome, which indicates the convincing changes in this protein after pathological treatment [50]. Peroxynitrite treatment of isolated beef heart mitochondria seems to induce the nitrosylation of cytochrome b-c1 complex subunit 1, but no such changes occurred in subunit 2 [51]. Proteomic studies of hepatic mitochondria in a mouse model of non-alcoholic fatty-liver disease revealed a decreased protein half-life of cytochrome b-c1 complex subunit 1, but not of subunit 2 [52]. Ischemia-reperfusion injury is associated with changes in cytochrome b-c1 complex subunit 1 [53]. Cytochrome b-c1 complex subunit 2 was abundantly expressed in high-litter-size spermatozoa, whereas cytochrome b-c1 complex subunit 1 was abundantly expressed in low-litter-size spermatozoa [54]. Accordingly, the expression of cytochrome b-c1 complex subunit 1 correlates negatively with litter size, whereas the expression of subunit 2 is proportional to litter size [54]. All these data indicate that these two subunits of the cytochrome b-c1 complex have different and specific roles in the functioning of complex III and that their changes are not often correlated with each other. Previously, we assessed the expression of yet another subunit, cytochrome b-c1 complex subunit 5, in reference to GAPDH and did not find any significant differences among the hippocampi and neocortices of WT and *Mecp2^−/y^* mice [21].

Our metabolomics data indicate increased OXPHOS in the *Mecp2^−/y^* neocortex [18]. This might represent an attempt to compensate for the mitochondrial impairments and less-efficient respiration reported for RTT mice [15,25]. The increase in cytochrome b-c1 complex subunit 1 supports this assumption.

It is well documented that the mitochondrial respiratory complexes I, III2, and IV are able to assemble into different combinations, thereby forming supramolecular structures (so-called supercomplexes), which co-exist with unbound CIII2 and CIV [55]. The regulatory details of supercomplex formation, i.e., the structural reorganization of the mitochondrial respiratory chain by which OXPHOS function can be fine-adjusted to the respective energy demands, remain to be resolved [56]. Recently, two separate mitochondrial respiratory chain organizations, referred to as S-MRC and C-MRC, were confirmed to co-exist in human cells and postmitotic tissues, and apparently, this is based on the preferential expression of the three COX7A subunit isoforms COX7A1, COX7A2, and SCAFI [57]. We cannot exclude the possibility that the mitochondria of MeCP2-deficient brains are characterized by different supramolecular assemblies, which may determine the specific features of these organelles. At least in the neocortex, hippocampus, and cardiac tissue of adult *Mecp2^−/y^* mice, any obvious alterations in supercomplex assembly do not seem to be present [21]. In functional terms, a reduction of complex II + III enzyme activity was confirmed by others in the skeletal muscle of male *Mecp2*-mutant mice, but the corresponding changes in enzyme activity were not detectable in the whole brain, cerebellum, and hippocampus of these mice [16].

### 4.5. ATP Synthase Complex of the OXPHOS System

Our 2-D gels with subsequent MS analyses indicate an upregulation of ATP-synthase subunit d in the *Mecp2^−/y^* hippocampus. In contrast, we previously observed a decreased ratio of ATP-synthase subunit beta (ATP5B) and GAPDH in *Mecp2^−/y^* compared with the WT neocortex [21]. Moreover, in symptomatic *Mecp2*-mutant female mice, a reduced activity of the ATP synthase FoF1 complex was observed [25]. The available data present a range of findings concerning the metabolic conditions of MeCP2-deficient brain tissue. Reduced brain ATP levels in *Mecp2*-null mice [17] and heterozygous *Mecp2*-mutant female mice [25], increased ATP production with intensified ATP turnover rates in neonatal *Mecp2^−/y^* hippocampal neurons [58], comparable ATP contents in acute hippocampal tissue slices from *Mecp2^−/y^* mice [59], and unchanged ATP/O ratios in male *Mecp2*-null mouse brain mitochondria [15] were reported. It, therefore, seems that more detailed analyses with cell-type-specific resolutions will be required to solve the issue of cellular ATP supply and consumption in MeCP2-deficient neuronal networks.

### 4.6. Modulatory and Metabolic Key Players

Our previous metabolomics study revealed that the *Mecp2^−/y^* neocortex has higher citrate amounts compared to WT mice, but no significant differences were detected for its two precursors, oxaloacetate and acetyl-CoA [18]. The present study did not reveal any differences between the citrate synthase activities in the neocortex of *Mecp2^−/y^* and WT mice. It is possible to suggest that the existing amount and/or activity of this enzyme in the *Mecp2^−/y^* neocortex is sufficient to produce increased amounts of the first product of the citric acid cycle according to the needs of a given cell type and its mitochondria.

The catalytic subunit alpha of the cAMP-dependent protein kinase is down-regulated in the *Mecp2^−/y^* hippocampus and *Mecp2^−/y^* neocortex. This downregulation should decrease the cAMP-dependent phosphorylation of target proteins. Decreased phosphorylation of mitochondrial protein NDUFS4 by cAMP-dependent protein kinase was demonstrated in the cerebellum of MeCP2-deficient mice, and the authors suggested two different scenarios: Either a deficit of cAMP-mediated phosphorylation or a decreased level of the protein itself [25]. Our data point to a deficit in the cAMP-dependent phosphorylation system. This view is further supported by reports on lowered cAMP levels in the neonatal [60] and the adult brainstem of *Mecp2^−/y^* mice [61]. Others detected increased levels of the regulatory protein kinase A subunit in protein extracts of the hippocampal CA1/CA3 regions of *Mecp2^−/y^* mice but found no significant changes for the catalytic subunit [62]. More importantly, they reported a reinstatement of stable synaptic long-term potentiation in the *Mecp2^−/y^* hippocampus upon pharmacological block of cAMP degradation by rolipram [62].

Prohibitin 1 was identified as a significantly upregulated protein both in the hippocampus and neocortex of *Mecp2^−/y^* mice compared with WT tissues. Prohibitin 1 and prohibitin 2 exist in the cytosol, the nucleus, and mitochondria, but a critical role is assumed especially for mitochondria (reviewed in [63]). These two proteins associate with the inner mitochondrial membrane, giving rise to a supra-macromolecular structure, which regulates mitochondrial metabolism [63]. Prohibitins play a role in the activity regulation of the OXPHOS system by physically interacting with distinct subunits of the respiratory complexes and affecting their stability and translation [63]. It was shown that upon neuronal injury, the overexpression of prohibitin 1 intensifies complex I (NADH)-dependent mitochondrial respiration and promotes the generation of ATP [64]. In accordance, the downregulation of prohibitin 2 decreases the expression and activities of respiratory chain complexes and leads to mitochondrial fragmentation [65]. Our previous metabolomics studies indicated increased OXPHOS in the neocortex of *Mecp2^−/y^* mice. We, therefore, suggest that increased amounts of prohibitin 1 may contribute to this process.

Creatine kinase B was identified as an upregulated protein in the mitochondrial fraction of the *Mecp2^−/y^* hippocampus, and the observed differences were further confirmed by Western blotting. Creatine kinase B is a pivotal player in cerebral energy homeostasis, and it also mediates crucial effects in GABAergic neurons by stimulating the neuronal K^+^/Cl^-^ cotransporter KCC2 [66]. Epigenomic analyses were conducted on RTT-diagnosed monozygotic twins, who presented different disease manifestations and clinical severities even though they shared an identical *MECP2* mutation and X-chromosome inactivation pattern [67]. The dermal fibroblasts obtained from these patients showed differences in DNA methylation patterns within the upstream regions of different genes contributing to brain function, including creatine kinase B. As predicted, the extent of DNA methylation in these upstream regions was inversely proportional to the level of gene expression [67]. However, in this study, no controls were used, and it is not clear if creatine kinase B increased or decreased compared to healthy controls. Nevertheless, the increased amount of creatine kinase B observed in the *Mecp2^−/y^* hippocampus of our mice could reflect such compensatory changes aiming to improve brain energy homeostasis.

14-3-3 protein theta, according to the 2-D electrophoresis experiments, is downregulated in the *Mecp2^−/y^* neocortex, and this finding was validated by further Western immunoblotting experiments. The 14-3-3 proteins represent a family of highly conserved scaffolding proteins, which are present in all eukaryotes, control the function/activity of a broad range of other cellular proteins, and thereby contribute to crucial cellular processes including energy metabolism (reviewed in [68]). The upregulation of the 14-3-3 protein improves mitochondrial function under the conditions of disruptive redox status and an impaired electron transfer pathway [69]. We, therefore, suggest that the observed downregulation of 14-3-3 theta in the *Mecp2^−/y^* mouse neocortex may contribute to the altered mitochondrial functioning in RTT.

Gamma enolase is downregulated both in the neocortex and hippocampus of *Mecp2^−/y^* mice. Enolase enzymes are cytosolic carbon-oxygen lyases, which are abundantly expressed and play a role in glucose metabolism. In vertebrates, different genes code for three isozymes of enolase: Alpha, beta, and gamma. Alpha enolase is ubiquitous, beta enolase is specific for muscles, and gamma enolase is specific for neurons [70]. Enolase has been shown recently to mediate various regulatory functions, beyond gluconeogenesis and glycolysis [71]. Our previous metabolomics studies showed an upregulation of five compounds related to glycolysis/gluconeogenesis processes [18]. Accordingly, the downregulation of one of the key enzymes of glycolysis processes seems unexpected. We have to take into account, however, that the downregulation of gamma enolase was detected in the isolated mitochondria of the neocortex and hippocampus of *Mecp2^−/y^* mice and that cytosolic fractions were not studied. Published data indicate the presence of enolase in the intermembrane space/outer mitochondrial membrane fraction [72]. Furthermore, enolase could play an additional specific role in mitochondrial functioning by transporting defined t-RNAs. In yeast, it was shown that only a small fraction of the lysine transfer RNA acceptor 1 (tRK1) pool (~3%) is translocated to the mitochondria, whereas the second isoacceptor, tRK2, resides in the cytosolic compartment [73]. The modulation of mitochondria by tRK1 is assumed to drive mitochondrial translation during the conditions of stress [73]. The mitochondrial import of tRK1 is facilitated by enolase 2 [73]. As an analogy, we could speculate that the downregulation of gamma enolase, in spite of the upregulation of glycolysis, serves another yet unidentified role of the enzyme in mitochondrial functioning.

## 5. Conclusions

Our data clearly show various alterations of the mitochondrial proteome in the neocortex and hippocampus of male MeCP2-deficient mice. These differences provide further insights into the special role of brain mitochondria in RTT-related pathology. The observation that the changes in protein expression show only partial overlap within the two brain regions emphasizes the importance of brain-region-specific analyses and detailed cross-regional comparison. Only then can the full picture of mitochondrial alterations in RTT be obtained.

Multiple mitochondria-relevant genes were found to be upregulated in transcriptional analyses on patient-derived peripheral blood samples [74]. Moreover, reviewing the RTT-related alterations across human-derived tissues identifies—in addition to dendritic/synaptic alterations and glial function—mitochondria as one of the three main targets affected by RTT [75]. Furthermore, whole-cell proteomics studies of the dermal fibroblasts from control and RTT patients have shown that the main changes occur in the expression of those proteins being part of the mitochondrial network [76]. Namely, ATP synthase subunit g, cytochrome c oxidase subunit 6B1, NADH-cytochrome b5 reductase 3, and mitochondrial peroxiredoxin-5 were found to be upregulated in RTT patients [76]. However, mitochondrial composition and bioenergetic features show a clear tissue specificity [77], and differences found in lymphocytes and fibroblasts may not necessarily be present in the brain tissue as well. Nevertheless, the upregulation of various components of the mitochondrial complexes in different tissues may be indicators of a diminished efficacy of mitochondrial respiration, possibly accompanied by an aberrant ATP production [74,75].

The brain is clearly not the only organ affected by mitochondrial alterations in RTT and certainly not the only tissue determining the clinical manifestation and the severity of this disorder. However, in view of its high metabolic demand and the absence of energy reserves, brain metabolism can be expected to be particularly struck by any type of mitochondrial alterations. In RTT-patient post-mortem brain tissue, the only change in the mitochondrial proteome reported so far is the downregulation of cytochrome c oxidase subunit I. This downregulation was observed, however, at the transcript level but not at the protein level [20]. Hence, the challenge will be to define the details of these alterations and pinpoint their functional consequences in different brain regions.

## Figures and Tables

**Table 1:** Comparison of the expression levels for selected proteins of the different OXPHOS components in hippocampus and neocortex of *Mecp2^−/y^* and WT mice. None of the differences were statistically significant (see *p*-values; degrees of freedom df = 10 for all comparisons; SD = standard deviation).

Protein	Hippocampus			Neocortex		
	Relative Units (Mean ± SD)		*p*-Value	Relative Units (Mean ± SD)		*p*-Value
	WT	*Mecp2^−/y^*		WT	*Mecp2^−/y^*	
NADH: ubiquinone oxidoreductase subunit B8 (NDUFB8), complex I	1.35 ± 0.11	1.17 ± 0.23	0.12	0.78 ± 0.15	0.85 ± 0.21	0.51
Succinate dehydrogenase complex iron sulfur subunit B (SDHB), complex II	0.44 ± 0.07	0.50 ± 0.17	0.42	0.87 ± 0.11	0.92 ± 0.16	0.7
Ubiquinol-cytochrome C reductase core protein 2 (UQCRC2, alternate name cytochrome B-C1 complex subunit 2), complex III	0.44 ± 0.16	0.53 ± 0.16	0.32	1.05 ± 0.11	1.06 ± 0.13	0.9
Cytochrome c oxidase subunit 1 (MTCO1), complex IV	0.27 ± 0.04	0.29 ± 0.05	0.43	0.87 ± 0.13	0.85 ± 0.16	0.84
ATP synthase F1 subunit alpha (ATP5A), FoF1 ATP synthase complex	0.61 ± 0.12	0.68 ± 0.20	0.73	1.11 ± 0.08	1.09 ± 0.13	0.74

**Table 2:** Differentially expressed proteins identified in the mitochondrial proteomes of *Mecp2^−/y^* and WT mice. In the experiments, we selected protein spots differing at least 2-fold between *Mecp2^−/y^* and WT mice. The data of those spots coinciding with location (pI, molecular weight) and protein identity in the different experiments underwent two-tailed Student´s *t*-tests (significance level 5%) to rate significant genotype-related differences. For each protein, Uniprot identifier, molecular/biological function, and %Volume of the spots (%Vol, expressed as mean ± SD) are listed. Degrees of freedom for genotypic comparison of the listed proteins are 4–10.

N	Protein	Direction of Changes as Compared to WT	Gene	Uniprot ID	% Vol Wt Sample, *Mecp2^-/Y^* Sample, *p*-Value	Molecular/Biological Functions of Proteins
**HIPPOCAMPUS**						
1	Pyruvate dehydrogenase E1 component subunit beta, mitochondrial	upregulated	*Pdhb*	Q9D051 ODPB_MOUSE	0.24 ± 0.23 (n = 6) 0.68 ± 0.17 (n = 6) *p* = 0.004	pyruvate dehydrogenase (acetyl-transferring) activity/glucose metabolic process
2	NADH-ubiquinone oxidoreductase 75 kDa subunit, mitochondrial	upregulated	*Ndufs1*	Q91VD9 NDUS1_MOUSE	0.20 ± 0.14 (n = 6) 0.53 ± 0.14 (n = 6) *p* = 0.002	2 iron, 2 sulfur cluster binding/electron transfer activity
3	NADH dehydrogenase [ubiquinone] iron- sulfur protein 8, mitochondrial	upregulated	*Ndufs8*	Q8K3J1 NDUS8_MOUSE	0.46 ± 0.41 (n = 6) 1.24 ± 0.66 (n = 6) *p* = 0.034	NADH dehydrogenase (ubiquinone) activity/mitochondrial electron transfer, NADH to ubiquinone
4	NADH dehydrogenase [ubiquinone] flavoprotein 2, mitochondrial	upregulated	*Ndufv2*	Q9D6J6 NDUV2_MOUSE	0.066 ± 0.075 (n = 4) 0.36 ± 0.12 (n = 4) *p* = 0.006	NADH dehydrogenase (ubiquinone) activity/mitochondrial electron transfer, NADH to ubiquinone
5	Cytochrome b-c1 complex subunit 1, mitochondrial	upregulated	*Uqcrc1*	Q9CZ13 QCR1_MOUSE	0.22 ± 0.20 (n = 6) 0.61 ± 0.23 (n = 6) *p* = 0.01	metal ion binding/mitochondrial electron transfer, ubiquinol to cytochrome c
6	ATP synthase subunit d	upregulated	*Atp5pd*	Q9DCX2 ATP5H_MOUSE	0.16 ± 0.12 (n = 6) 0.61 ± 0.25 (n = 6) *p* = 0.002	proton-transporting ATP synthase activity, rotational mechanism/proton motive force-driven mitochondrial ATP synthesis
7	Creatine kinase B-type	upregulated	*Ckb*	Q04447 KCRB_MOUSE	0.23 ± 0.17 (n = 6) 0.92 ± 0.70 (n = 6) *p* = 0.038	Kinase activity/phosphocreatine biosynthetic process
8	Prohibitin 1	upregulated	*Phb*	P67778 PHB1_MOUSE	0.036 ± 0.031 (n = 4) 0.23 ± 0.10 (n = 4) *p* = 0.011	protein heterodimerization activity/mitochondrial organization
9	Gamma-enolase	downregulated	*Eno2*	P17183 ENOG_MOUSE	0.92 ± 0.27 (n = 6) 0.36 ± 0.26 (n = 6) *p* = 0.004	Lyase/glycolysis
10	cAMP-dependent protein kinase catalytic subunit alpha	downregulated	*Prkaca*	P05132 KAPCA_MOUSE	0.17 ± 0.08 (n = 3) 0.037 ± 0.008 (n = 3) *p* = 0.046	Serine/threonine protein kinase activity
NEOCORTEX						
11	Cytochrome b-c1 complex subunit 1, mitochondrial	upregulated	*Uqcrc1*	Q9CZ13 QCR1_MOUSE	0.07 ± 0.04 (n = 5) 0.20± 0.10 (n = 5) *p* = 0.039	metal ion binding/mitochondrial electron transfer, ubiquinol to cytochrome c
12	Guanine nucleotide-binding protein G(o) subunit alpha	upregulated	*Gnao1*	P18872 GNAO_MOUSE	0.20 ± 0.22 (n = 6) 0.70 ±0.47 (n = 6) *p* = 0.043	G protein-coupled receptor binding/G protein coupled receptor signaling pathway
13	Prohibitin 1	upregulated	*Phb*	P67778 PHB1_MOUSE	0.13 ± 0.09 (n = 6) 0.25 ± 0.05 (n = 6) *p* = 0.024	protein heterodimerization activity/mitochondria lorganization
14	Gamma-enolase	downregulated	*Eno2*	P17183 ENOG_MOUSE	0.48 ± 0.13 (n = 3) 0.11 ± 0.03 (n = 3) *p* = 0.009	Lyase/glycolysis
15	cAMP-dependent protein kinase catalytic subunit alpha	downregulated	*Prkaca*	P05132 KAPCA_MOUSE	0.28 ± 0.03 (n = 3) 0.11 ± 0.09 (n = 3) *p* = 0.031	Serine/threonine protein kinase activity
16	14-3-3 protein theta	downregulated	*Ywhag*	P61982 1433G_MOUSE	0.58 ± 0.15 (n = 4) 0.26 ± 0.14 (n = 4) *p* = 0.022	protein domain specific binding/protein targeting

## Data Availability

Data are contained within the article.

## Supplementary Materials

The following supporting information can be downloaded at: https://www.mdpi.com/article/10.3390/biology12070956/s1, Figure S1: Original Western blot images; Figure S2: Silver stained 2-D gels; Table S1: MS Data.
