# Supplementary material for: Mitochondrial Proteome Changes in Rett Syndrome
Source: Biology (Basel). 2023 Jul 3;12(7):956. doi: 10.3390/biology12070956 (PMC10376342; doi:10.3390/biology12070956)

Complete gel, Figure 2A (OXPHOS Complexes, Hippocampus)

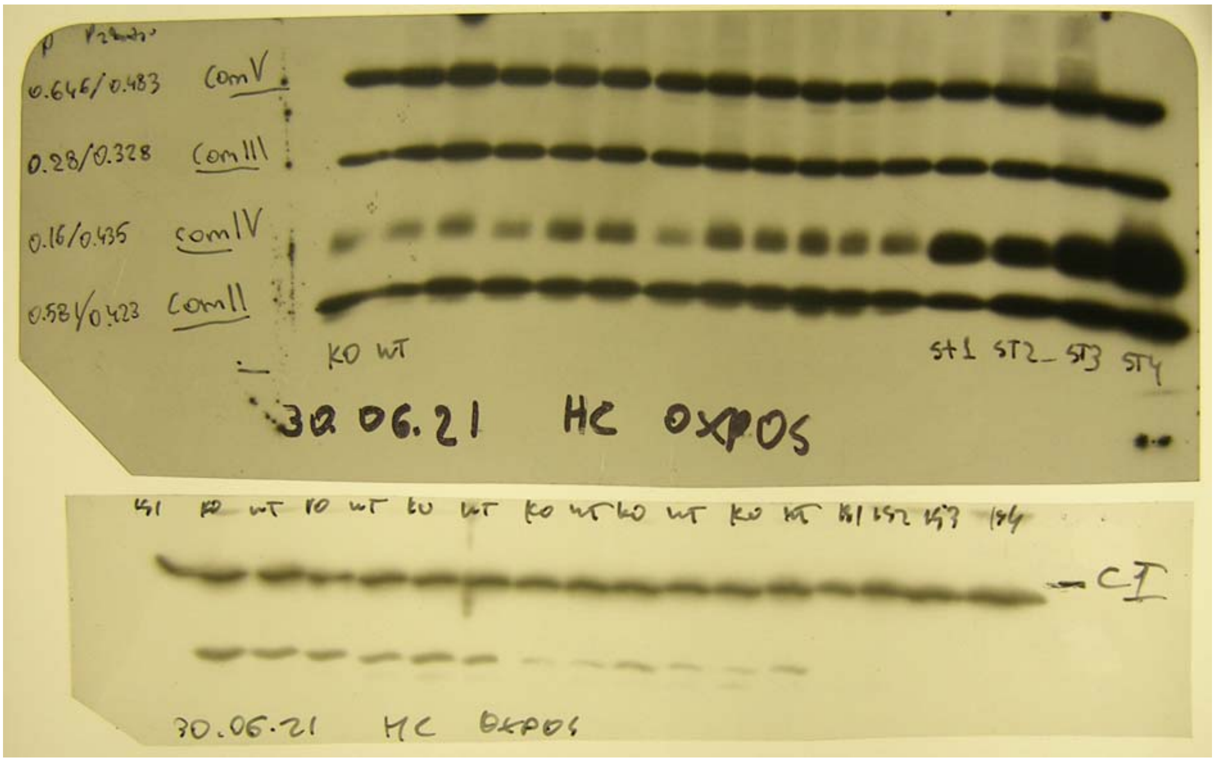

Complete gel, Figure 2B (OXPHOS complexes, Neocortex)

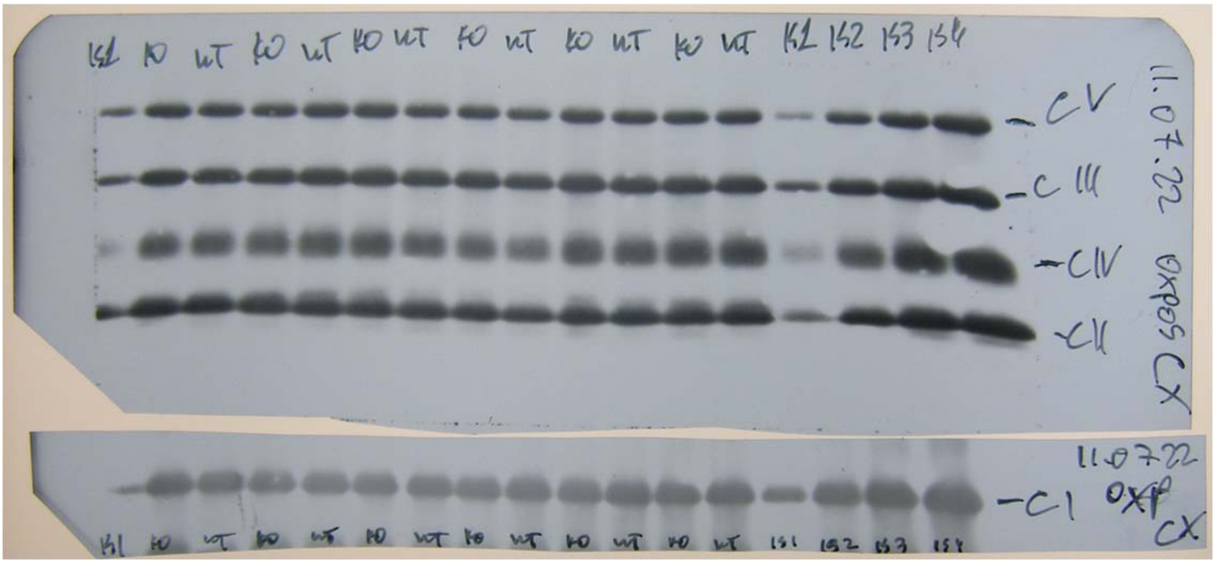

Complete gel, Figure 3A (Mitofusin-1, Neocortex)

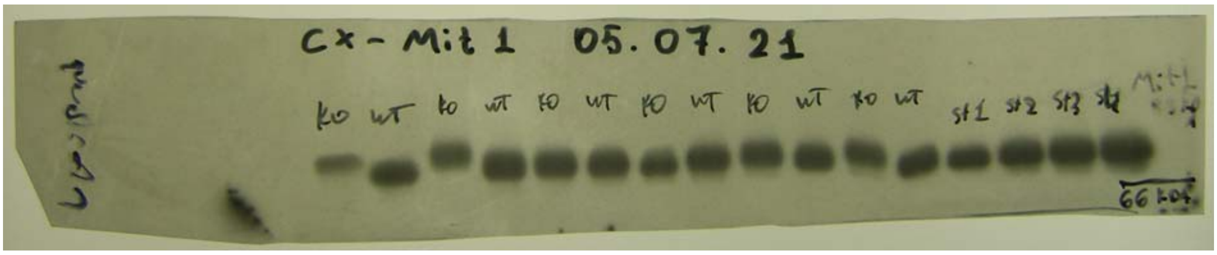

Complete gel, Figure 4A (DRP-1, Hippocampus)

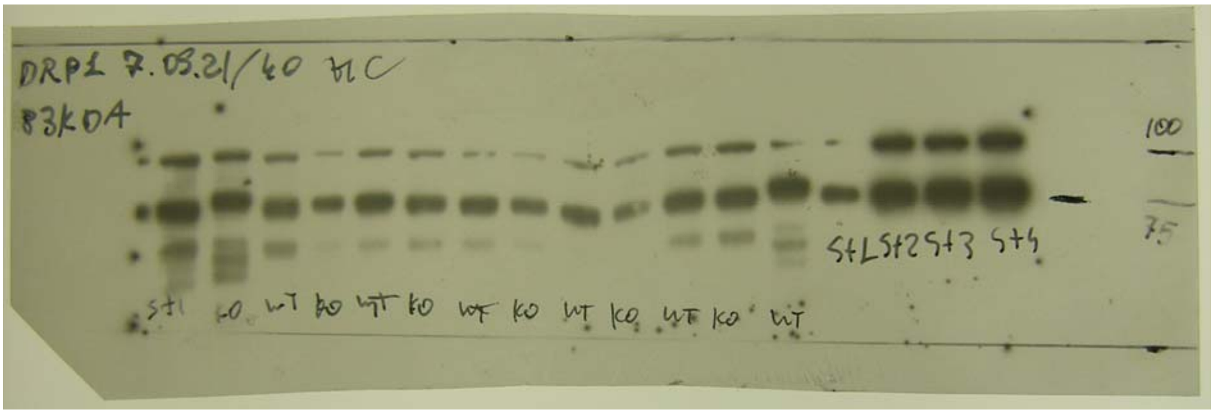

Gels shown in Figure 5A & 5B in the manuscript already do represent the complete gel images.

Complete gel, Figure 6A (Creatine kinase B, Hippocampus)

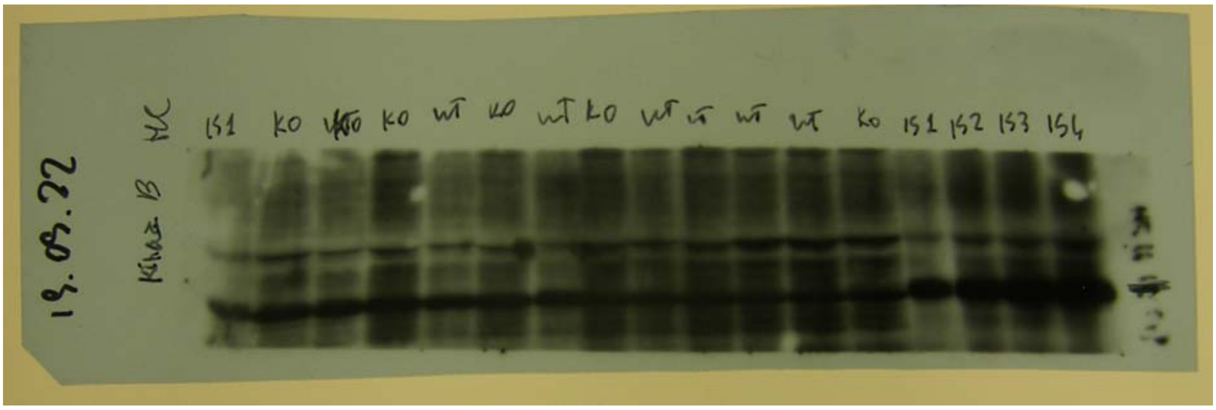

Complete gel, Figure 7A (14-3-3 Protein theta, Neocortex)

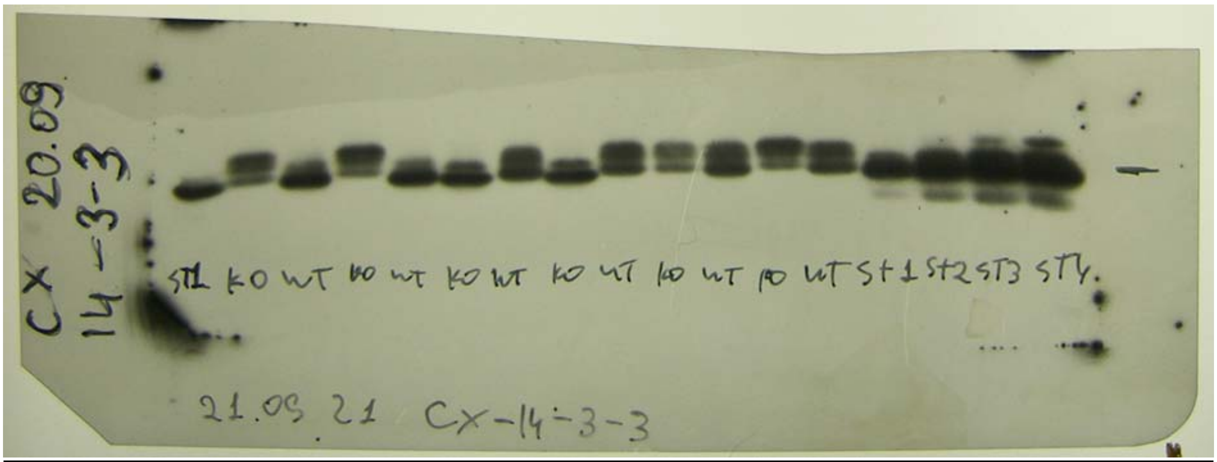

Complete gel, Figure 8A (HSP 60, Neocortex)

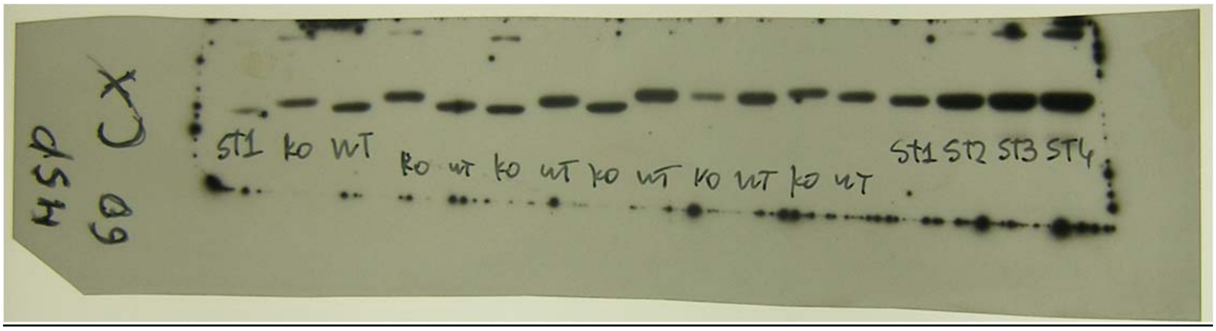

Supplement: Supplementary file 1 [file biology-12-00956-s001.zip › Figure S1. Original Western blot images_proofread.pdf]
