# Supplementary material for: Mitochondrial Proteome Changes in Rett Syndrome
Source: Biology (Basel). 2023 Jul 3;12(7):956. doi: 10.3390/biology12070956 (PMC10376342; doi:10.3390/biology12070956)
Supplement: Supplementary file 1 [file biology-12-00956-s001.zip › Figure S2. Silver stained 2-D gels_proofread.pdf]

Wildtype hippocampus

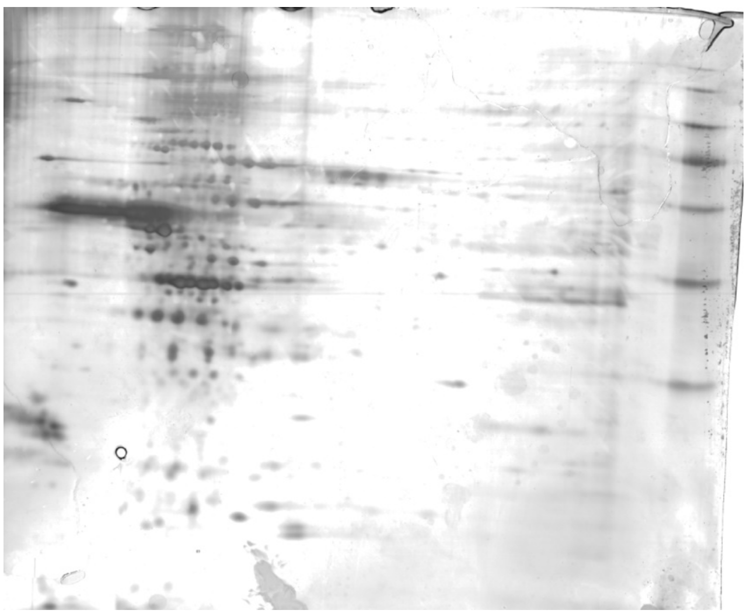

Wildtype hippocampus 1

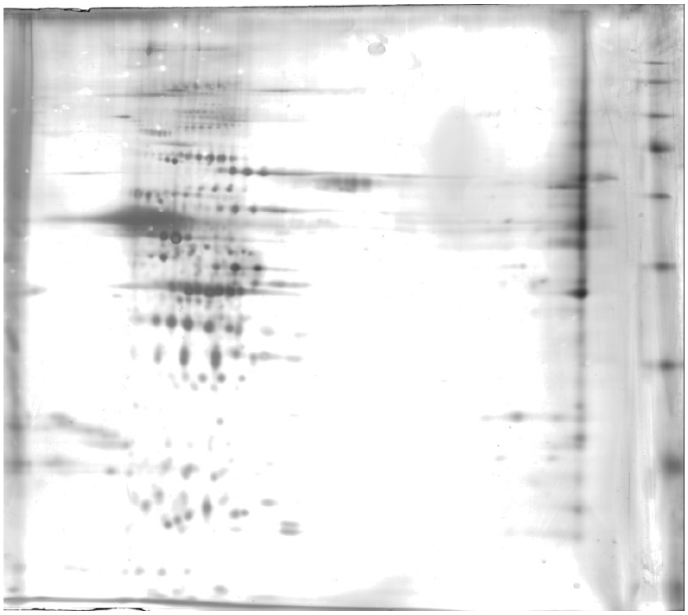

Wildtype hippocampus 2

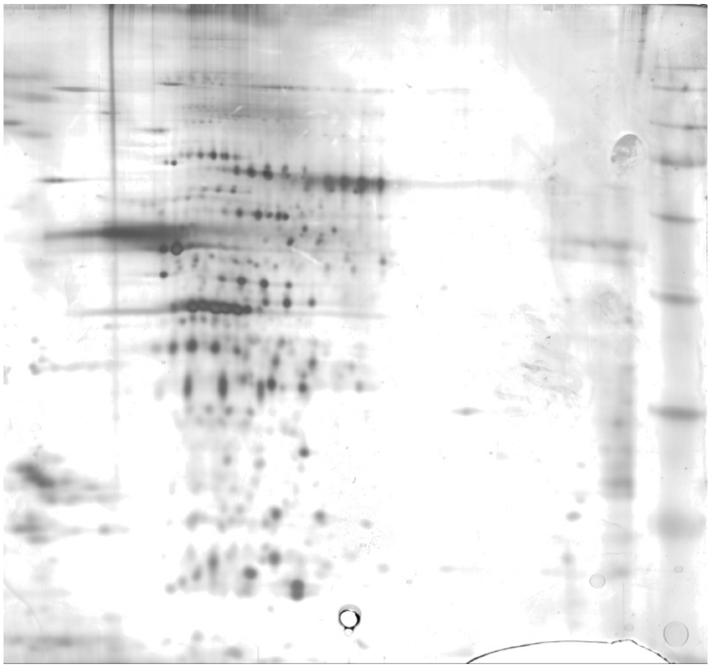

Wildtype hippocampus 3

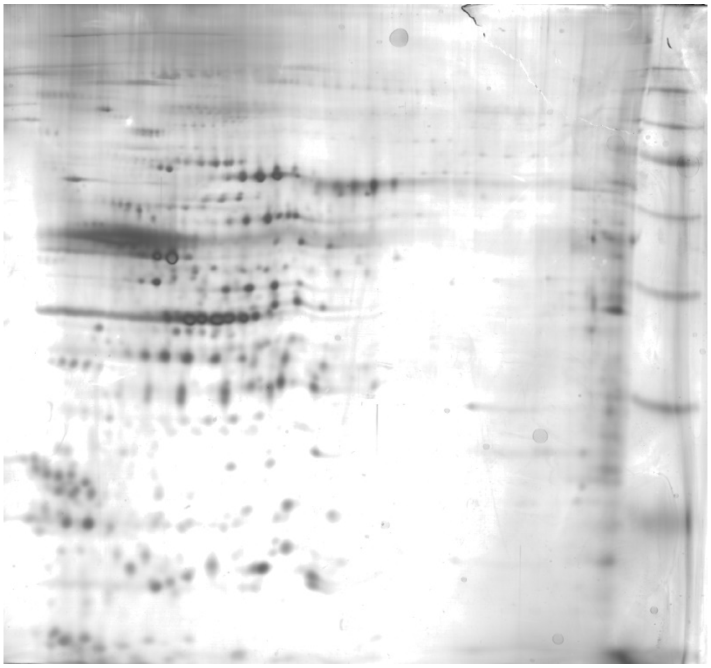

Wildtype hippocampus 4

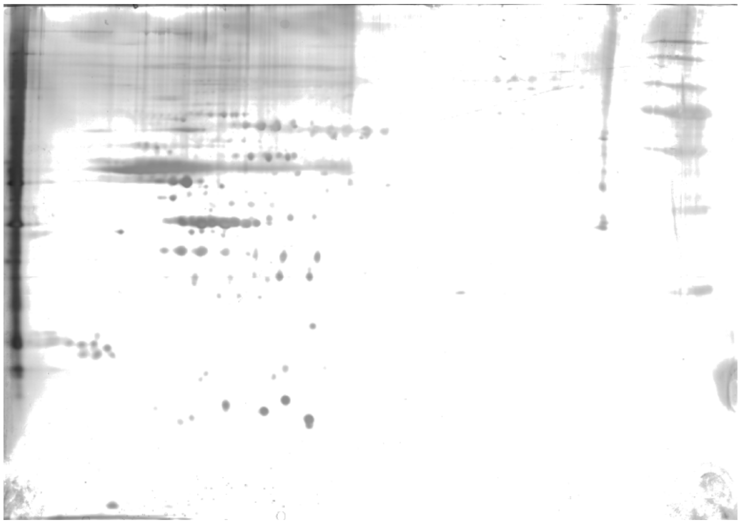

Wildtype hippocampus 5

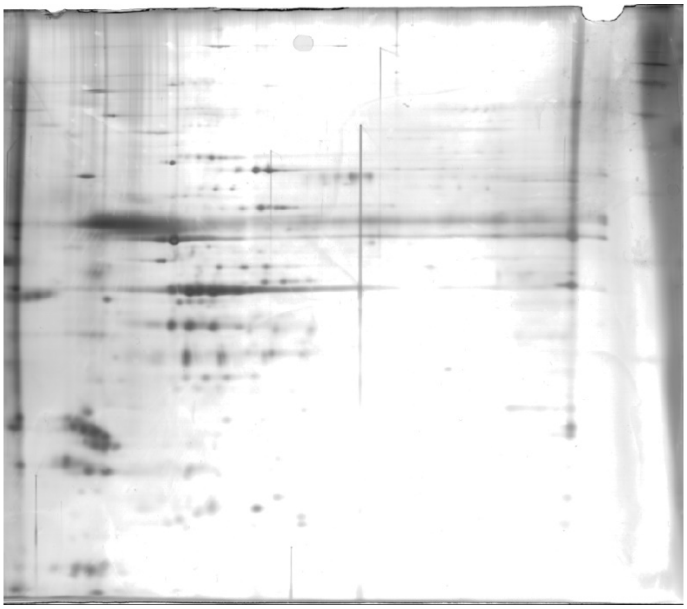

Wildtype hippocampus 6

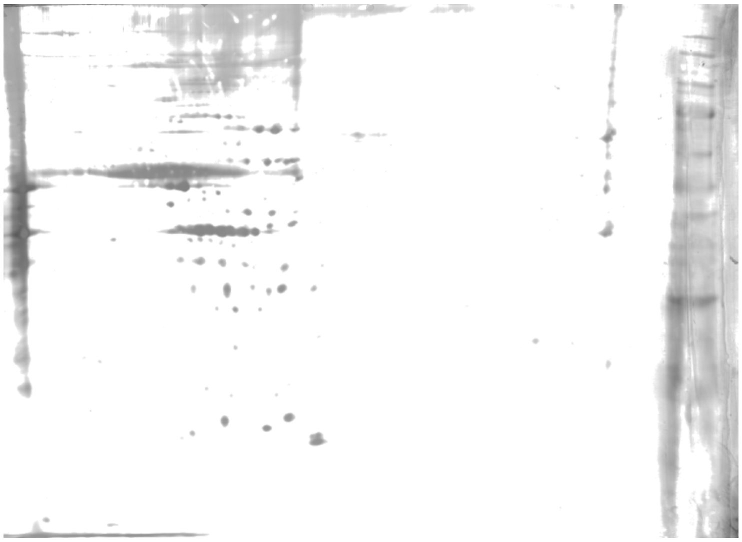

Wildtype hippocampus 7

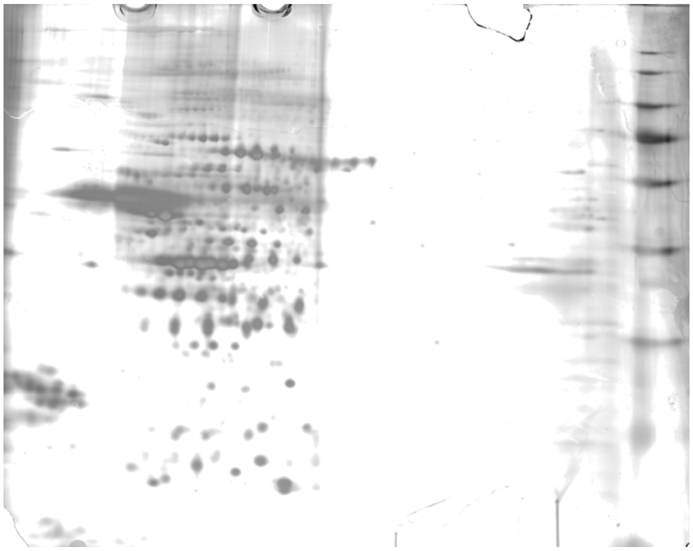

Wildtype hippocampus 8

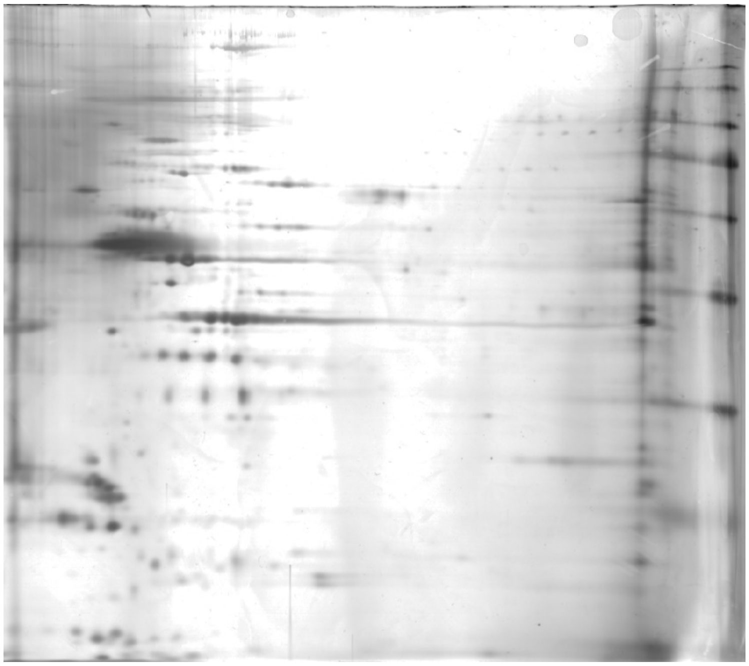

Wildtype hippocampus 9

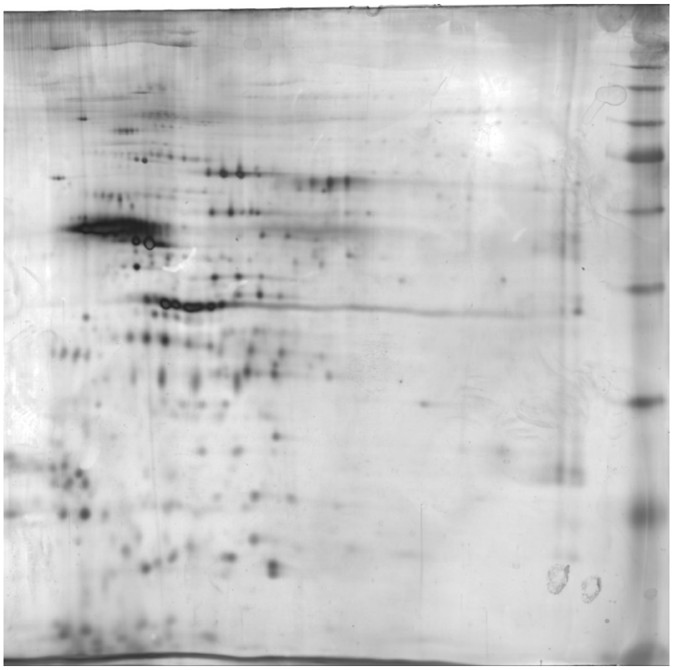

Wildtype hippocampus 10

Mecp2$^{-/y}$ **hippocampus**

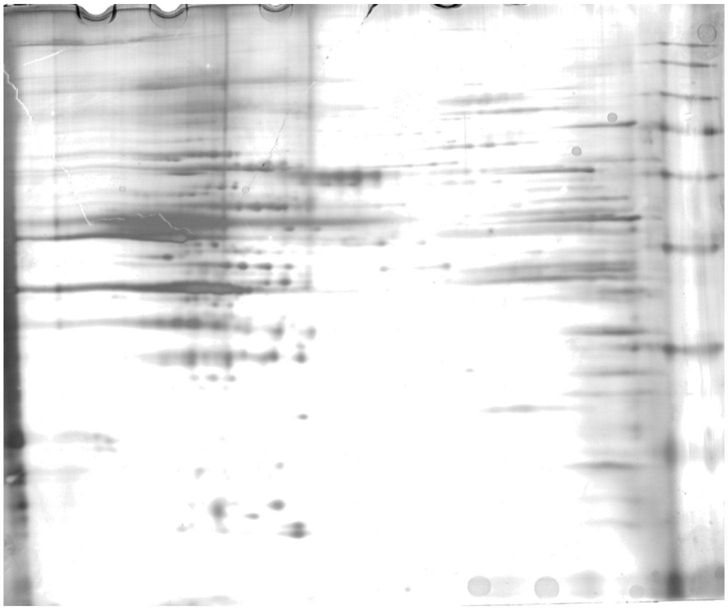

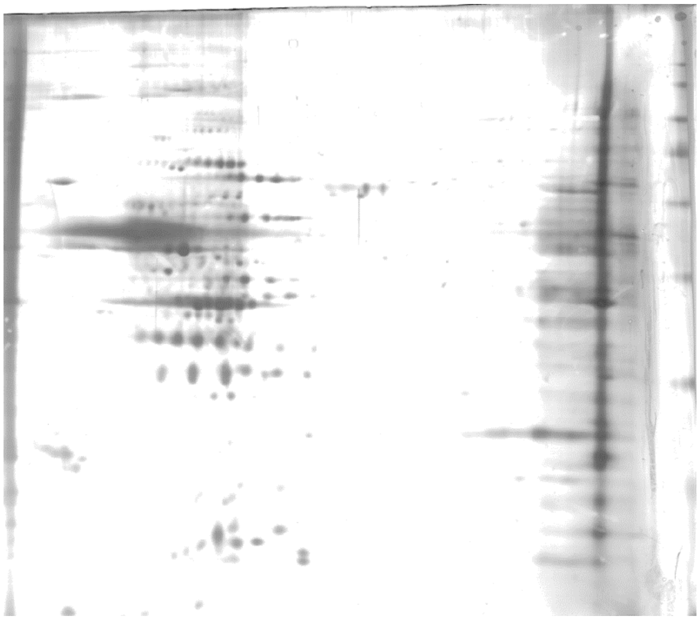

Mecp2$^{-/y}$ hippocampus 1

Mecp2$^{-/y}$ hippocampus 2

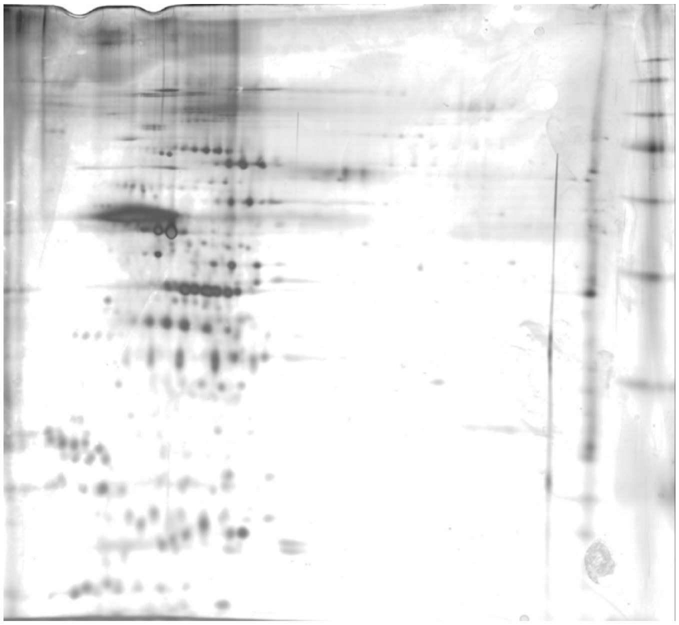

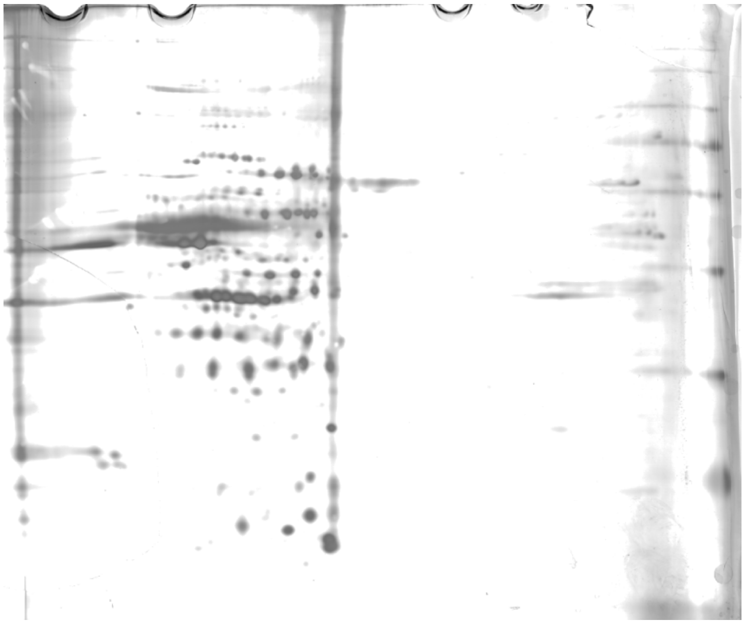

Mecp2$^{-/y}$ hippocampus 3

Mecp2$^{-/y}$ hippocampus 4

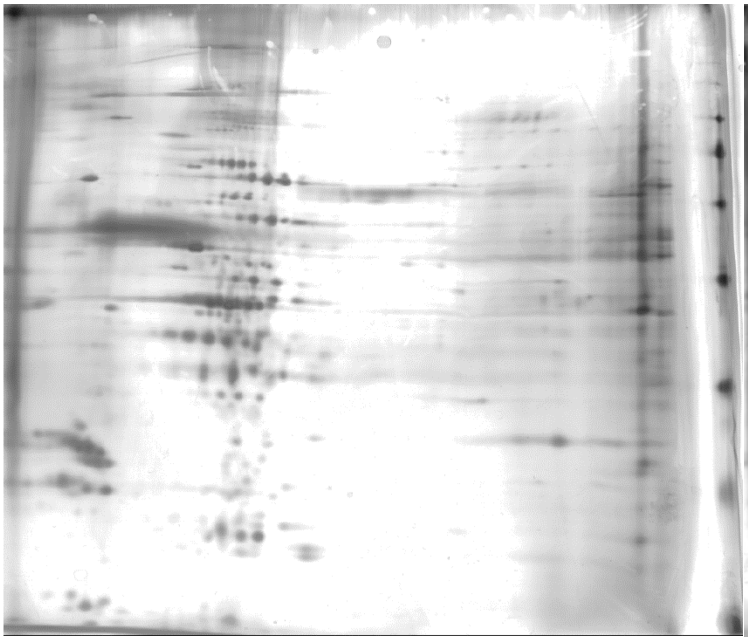

Mecp2$^{-/y}$ hippocampus 5

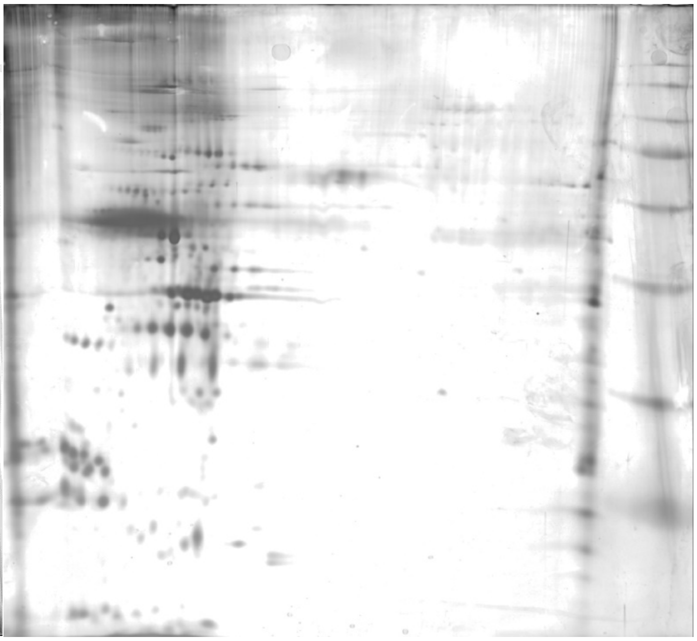

Mecp2$^{-/y}$ hippocampus 6

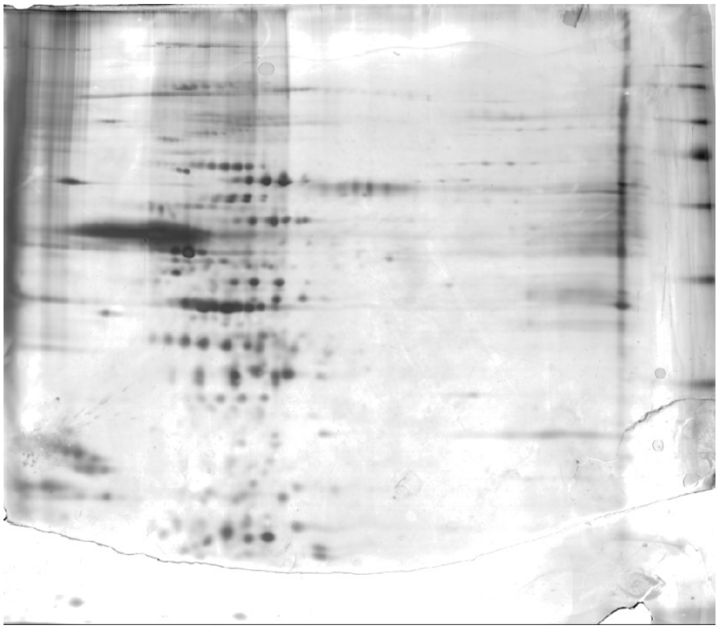

Mecp2$^{-/y}$ hippocampus 7

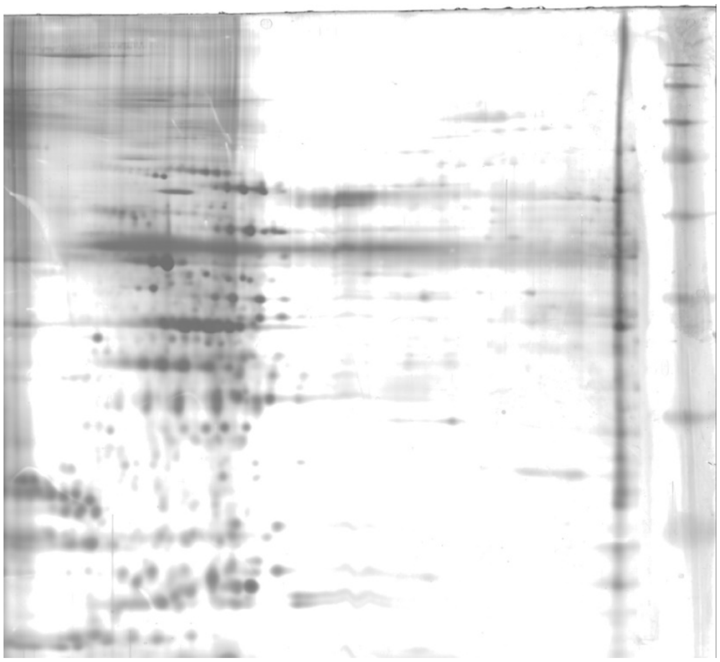

Mecp2$^{-/y}$ hippocampus 8

Wildtype neocortex

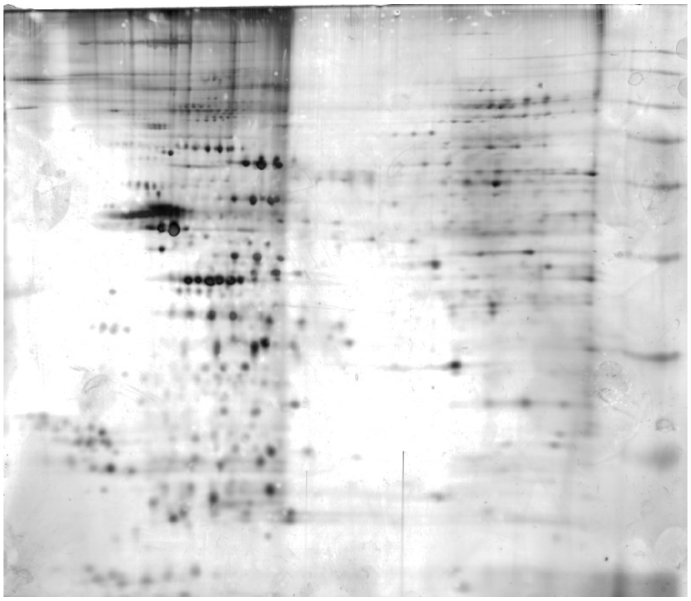

Wildtype neocortex 1

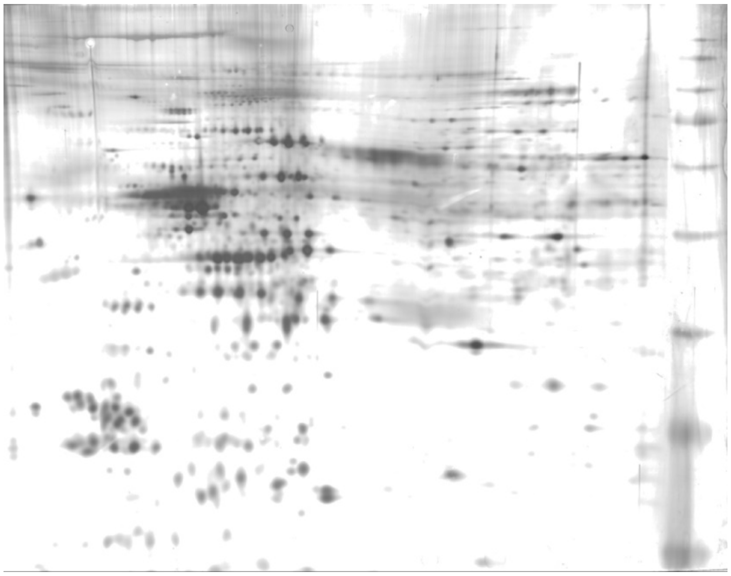

Wildtype neocortex 2

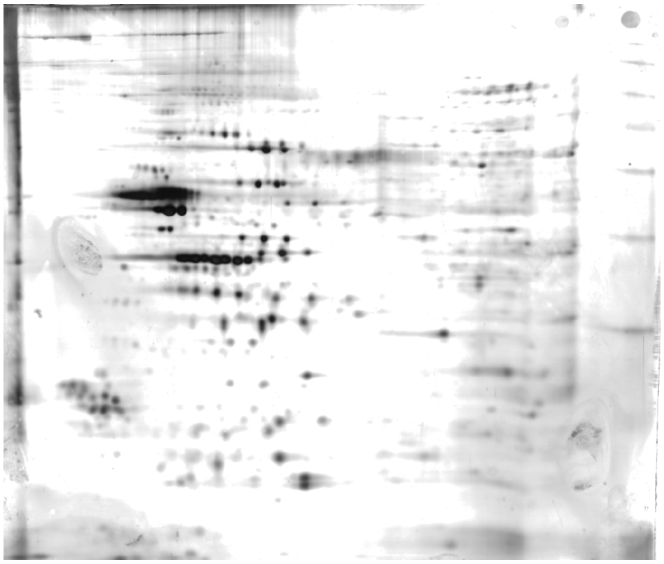

Wildtype neocortex 3

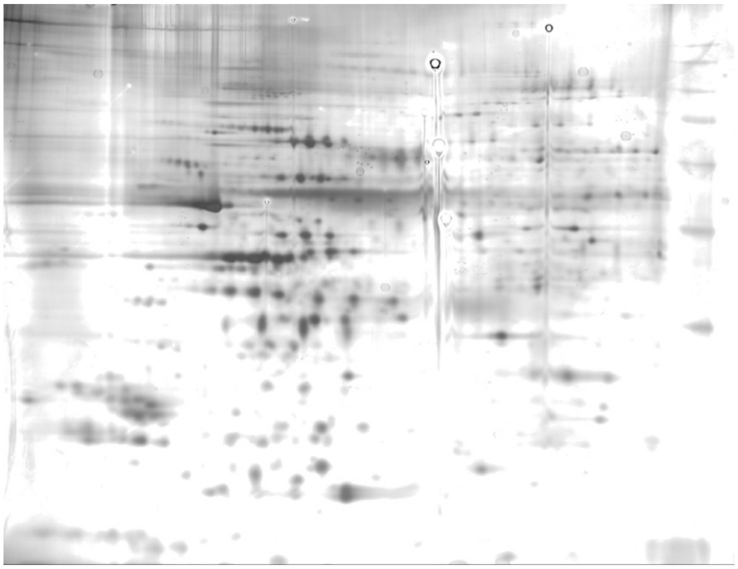

Wildtype neocortex 4

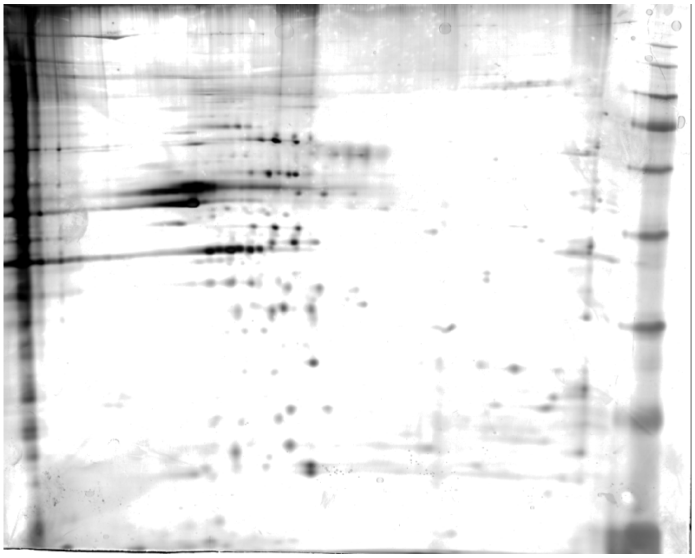

Wildtype neocortex 5

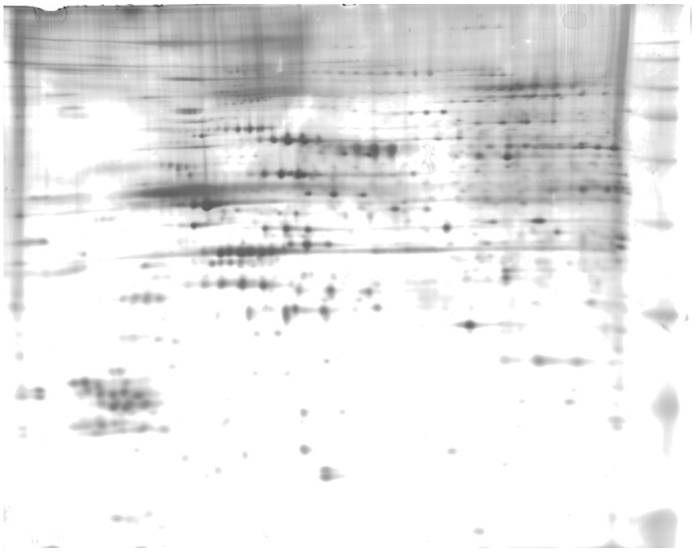

Wildtype neocortex 6

Mecp2$^{-/y}$ **neocortex**

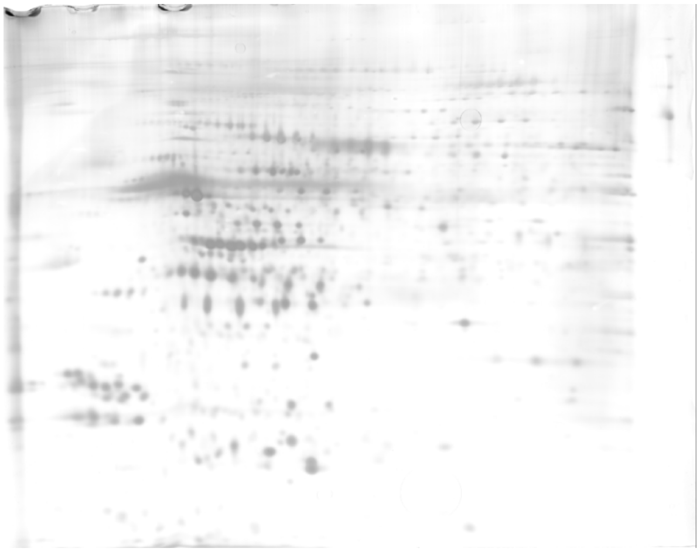

Mecp2$^{-/y}$ neocortex 1

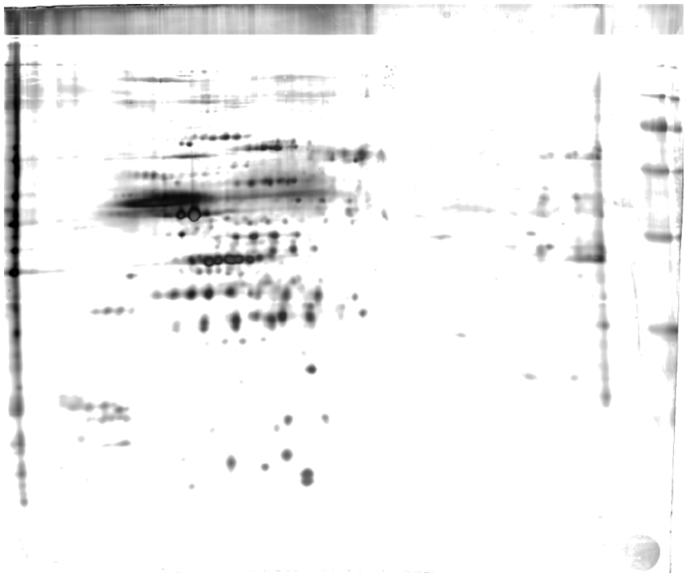

Mecp2$^{-/y}$ neocortex 2

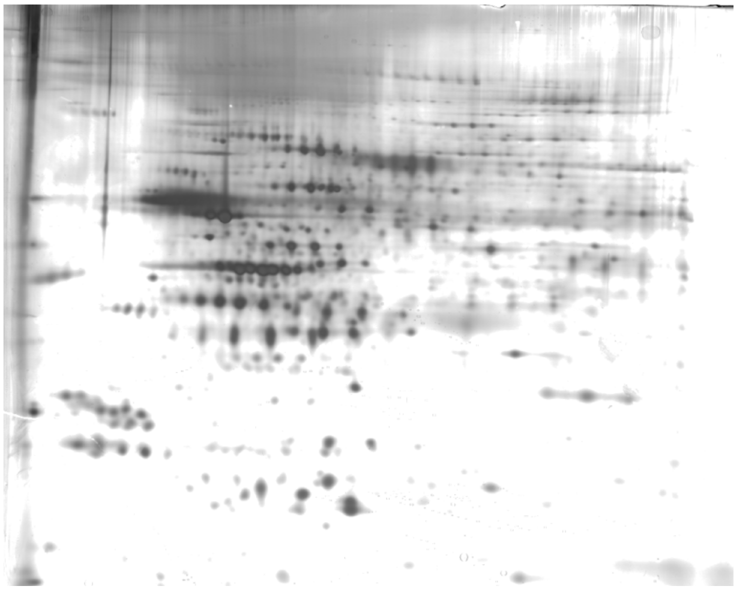

Mecp2$^{-/y}$ neocortex 3

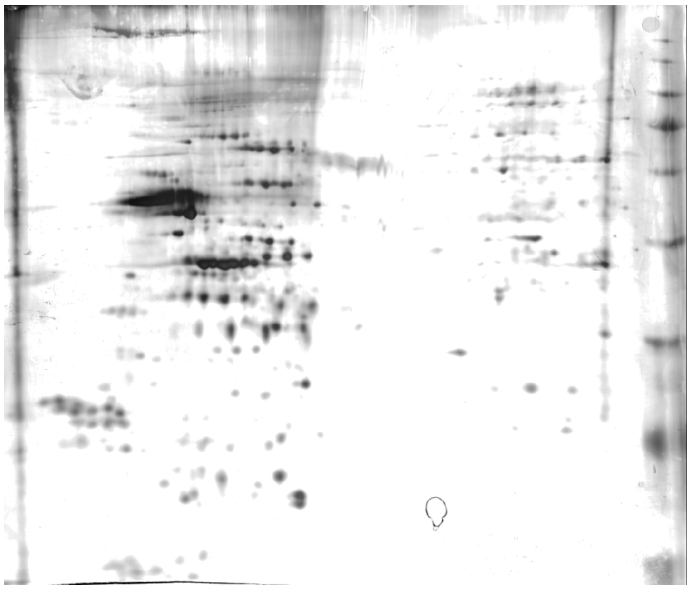

Mecp2$^{-/y}$ neocortex 4

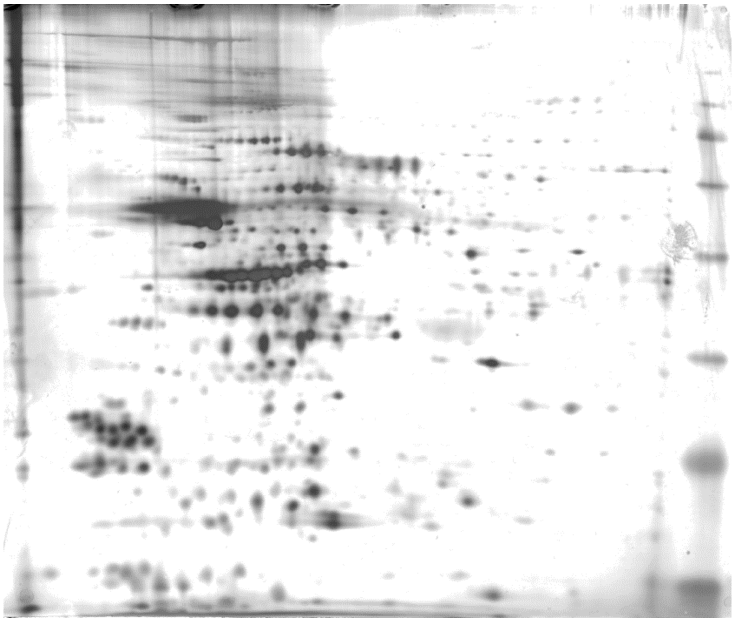

Mecp2$^{-/y}$ neocortex 5

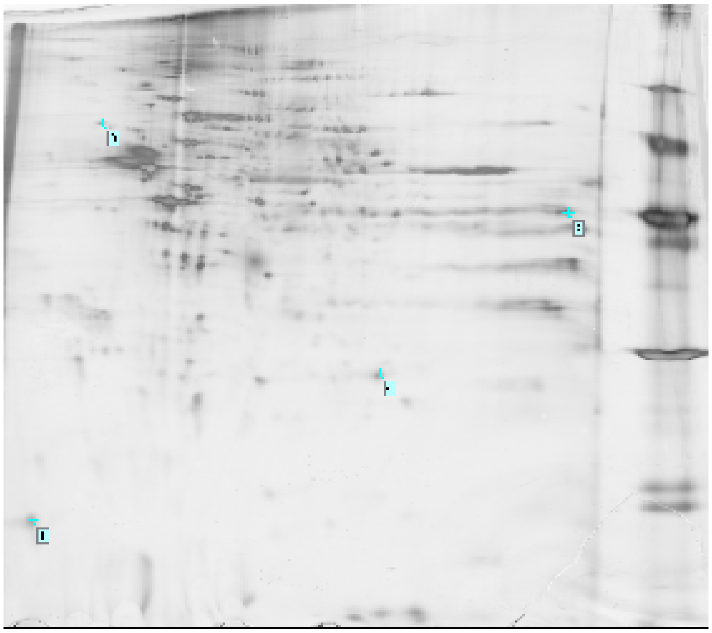

Mecp2$^{-/y}$ neocortex 6
