# Supplementary material for: Mitochondrial Proteome Changes in Rett Syndrome
Source: Biology (Basel). 2023 Jul 3;12(7):956. doi: 10.3390/biology12070956 (PMC10376342; doi:10.3390/biology12070956)
Supplement: Supplementary file 1 [file biology-12-00956-s001.zip › Table S1-MS Data_proofread.pdf]

Table S1. Representative data of proteins identified in spots by MS analysis.

HIPPOCAMPUS

AN	Protein	Score	Coverage	Proteins	Unique peptides	Peptides	Peptide spectrum matches (PSM)*	Amino acids	Molecular weight (KDa)	Calculated Pi
Q9D051	Pyruvate dehydrogenase E1 component subunit beta, mitochondrial	43.08	24.23%	1	6	6	18	359	38.9	6.87
Q9D051	Pyruvate dehydrogenase E1 component subunit beta, mitochondrial	12.16	8.91%	1	2	2	7	359	38.9	6.87
Q9D051	Pyruvate dehydrogenase E1 component subunit beta, mitochondrial	8.35	19.50%	1	5	5	7	359	38.9	6.87
Q9D051	Pyruvate dehydrogenase E1 component subunit beta, mitochondrial	4.92	7.52%	1	2	2	4	359	38.9	6.87
Q9D051	Pyruvate dehydrogenase E1 component subunit beta, mitochondrial	4.38	27.30%	1	7	7	10	359	38.9	6.87
Q91VD9	NADH-ubiquinone oxidoreductase 75 kDa subunit, mitochondrial	7.07	3.99%	1	2	2	3	727	79.7	5.72
Q91VD9	NADH-ubiquinone oxidoreductase 75 kDa subunit, mitochondrial	2.1	2.20%	1	1	1	1	727	79.7	5.72
Q8K3J1	NADH dehydrogenase [ubiquinone] iron-sulfur protein 8	21.86	8.49%	1	1	1	7	212	24	6.21
Q9D6J6	NADH dehydrogenase [ubiquinone] flavoprotein 2, mitochondrial	4.38	27.02%	1	3	3	6	248	27.3	7.4
Q9D6J6	NADH dehydrogenase [ubiquinone] flavoprotein 2, mitochondrial	20.79	51.61%	1	8	8	13	248	27.3	7.4

Q9CZ13	Cytochrome b-c1 complex subunit 1, mitochondrial	67	31.87%	1	10	10	23	480	52.8	6.21
Q9DCX2	ATP synthase subunit d, mitochondrial	5.69	38.51%	1	4	4	5	161	18.7	5.69
Q9DCX2	ATP synthase subunit d, mitochondrial	5.77	10.56%	1	1	1	3	161	18.7	5.69
Q04447	Creatine kinase B-type	32.03	19.95%	1	5	5	16	381	42.7	5.67
Q04447	Creatine kinase B-type	39.52	24.67%	1	7	7	15	381	42.7	5.67
Q04447	Creatine kinase B-type	55.27	17.32%	1	5	5	20	381	42.7	5.67
Q04447	Creatine kinase B-type	1.73	13.91%		3	3	3	381	42.7	5.67
P67778	Prohibitin-1	8.83	15.81%	1	3	3	6	272	29.8	5.76
P17183	Gamma-enolase	2.2	7.14%	1	1	1	1	434	47.3	5.11
P17183	Gamma-enolase	23.96	26.73%	3	8	8	21	434	47.3	5.11
P05132	cAMP-dependent protein kinase catalytic subunit alpha	17.63	3.70%	1	1	1	8	351	40.5	8.79

NEOCORTEX

**AN**	**Protein**	**Score**	**Coverage**	**Proteins**	**Unique peptides**	**Peptides**	**Peptide spectrum matches (PSM)***	**Amino acids**	**Molecular weight (KDa)**	**Calculated Pi**
Q9CZ13	Cytochrome b-c1 complex subunit 1, mitochondrial	12.98	21.25%	1	8	8	14	480	52.8	6.21
P18872	Guanine nucleotide-binding protein G(o) subunit alpha	7.86	7.63%	4	2	2	5	354	40.1	5.53
P18872	Guanine nucleotide-binding protein G(o) subunit alpha	28.46	14.97%	4	4	4	11	354	40.1	5.53
P18872	Guanine nucleotide-binding protein G(o) subunit alpha	14.81	26.55%	11	8	8	17	354	40.1	4.82
P18872	Guanine nucleotide-binding protein G(o) subunit alpha	6.7	21.47%	11	6	6	10	354	40.1	5.53

P67778	Prohibitin-1	13.8	38.97%	1	9	9	19	272	29.8	5.76
P17183	Gamma-enolase	56.01	38.94%	3	10	10	22	434	47.3	5.11
P05132	cAMP-dependent protein kinase catalytic subunit alpha	5.79	3.70%	1	1	1	5	351	40.5	8.79
P68254	14-3-3 protein theta	7.38	15.10%	1	3	3	3	245	27.8	4.78

* PSM value may be higher than the number of peptides identified for high-scoring proteins, as peptides may be identified repeatedly.
